# Incidence of immunotherapy‐related hyperprogressive disease (HPD) across HPD definitions and cancer types in observational studies: A systematic review and meta‐analysis

**DOI:** 10.1002/cam4.6970

**Published:** 2024-02-24

**Authors:** Min Jeong Kim, Seung Pyo D. Hong, Yeonggyeong Park, Young Kwang Chae

**Affiliations:** ^1^ Department of Medicine Northwestern University Feinberg School of Medicine Chicago Illinois USA; ^2^ Robert H. Lurie Comprehensive Cancer Center Northwestern University Chicago Illinois USA

**Keywords:** antineoplastic agent resistance, immunotherapy, systematic review

## Abstract

**Background:**

While evidence of hyperprogressive disease (HPD) continues to grow, the lack of a consensual definition obscures a proper characterization of HPD incidence. We examined how HPD incidence varies by the tumor type or the type of definition used.

**Methods:**

We searched PubMed, Embase, the Cochrane Library of Systematic Reviews, and Web of Science from database inception to June 21, 2022. Observational studies reporting HPD incidence, in patients diagnosed with solid malignant tumors and treated with immune checkpoint inhibitors (ICI), were included. Random‐effects meta‐analyses were performed, and all statistical tests were 2‐sided.

**Results:**

HPD incidence was 12.4% (95% CI 10.2%–15.0%) with evidence of heterogeneity (*Q* = 119.32, *p* < 0.001). Meta‐regression showed that the risk of developing HPD was higher in patients with advanced gastric cancer (adjusted odds ratio [OR], 10.83; 95% CI, 2.14–54.65; *p* < 0.001), hepatocellular carcinoma (adjusted OR, 7.99; 95% CI, 1.68–38.13; *p* = 0.006), non‐small cell lung cancer (adjusted OR, 7.14; 95% CI, 1.58–32.29; *p* = 0.005), and mixed or other types (adjusted OR, 5.09; 95% CI, 1.12–23.14, *p* = 0.018) than in patients with renal cell carcinoma. Across definitions, HPD defined as a tumor growth kinetics ratio ≥ 2 (adjusted OR, 1.82; 95% CI, 1.08–3.07; *p* = 0.025) based on the Response Evaluation Criteria in Solid Tumors (RECIST) reported higher incidence than when HPD was defined as RECIST‐defined progressive disease and a change in the tumor growth rate (TGR) exceeding 50% (∆TGR > 50).

**Conclusions:**

The incidence of immunotherapy‐related HPD may vary across tumor types and definitions used, supporting the argument for a uniform and improved method of HPD evaluation for informed clinical decision‐making.

## 1 INTRODUCTION

Immune checkpoint inhibitors (ICI) have revolutionized cancer therapy, demonstrating potential for durable response in a subset of patients across multiple advanced tumor types. At the same time, the past decade of ICI therapy has witnessed novel patterns of response previously unobserved with standard regimens. In particular, a paradoxical acceleration in the rate of tumor growth termed hyperprogressive disease (HPD) has been of increasing interest due to evidence of potential implications on patient prognosis.

As radiological criteria for objective progression are based on an evaluation of therapeutic efficacy from treatment onset, they are not sufficient to distinguish between a naturally aggressive disease course and a therapy‐related change in the tumor growth kinetics. Thus, a consideration of the pretreatment kinetics of tumor growth has been critical to defining this phenomenon from its earliest descriptions in 2017. A scalar comparison of changes in the longitudinal tumor burden between the treatment period and the pretreatment counterpart, accordingly, has been key in developing approaches to evaluate HPD.

Several different metrics have been proposed to describe the magnitude of change in the tumor's growth rate before and after immunotherapy in patients experiencing HPD. At the most fundamental level, the difference between these metrics is how the tumor size is quantified—unidimensional and bidimensional measurements, as well as volume estimates based on these measurements—have been used as surrogates for the tumor burden. The tumor growth kinetics (TGK)^1^ calculates a unidimensional change in the tumor size per unit time based on the sum of the diameters of target lesions per Response Evaluation Criteria in Solid Tumours (RECIST). The tumor growth rate (TGR)^2^ utilizes linear measurements of RECIST to estimate a percentage change in the tumor volume per month, based on the assumption that the tumor is roughly spherical and follows an exponential model of growth. The “progression pace”^3^ measures a bidimensional change in the tumor size per unit time using the sum of the products of the greatest axial dimensions of target lesions according to the immune‐related response criteria (irRC); another key difference that arises from using irRC instead of RECIST is that new lesions are incorporated into the sum of the measurements.

Despite the growing body of literature on this phenomenon, HPD continues to lack a standardized definition. Consequently, various operationalizations in the method of HPD evaluation—from the choice of radiological criteria to the quantitative approach in describing the change in tumor growth kinetics—has made challenging a proper interpretation of data. While incidences range from 0% in renal cell carcinoma^4^ to 29% in head and neck squamous cell carcinoma,^1^ it is difficult to determine whether these varying estimates reflect differential tumor‐intrinsic behavior or arise from differences across current definitions of HPD.

A 2019 meta‐analysis evaluated evidence on HPD incidence and risk factors associated with the phenomenon, although it was limited to nine studies and therefore could not assess potential differences across tumor types or the types of definition used. Another systematic review and meta‐analysis, based only on NSCLC cohorts, reported on predictive clinicopathological factors of HPD and demonstrated an association between HPD patients and worse overall survival. This review included six studies in which no definitions were identical, and two definitions did not incorporate pretreatment tumor kinetics in the evaluation of HPD. The most recent and comprehensive systematic review and meta‐analysis—including a total of 24 studies—conducted subgroup analyses according to the tumor type and four categories of HPD definitions. However, these analyses were limited to two tumor types; additionally, the definitions were classified into broad categories, with varying thresholds and evaluation criteria within each category. The consistency in incidence estimates within and between definitions, which would make possible a robust comparison between tumor types, has yet been documented in any meta‐analytic study.

The aims of this systematic review were to describe and evaluate the current evidence on HPD across variable definitions integrating pretreatment tumor growth kinetics and to investigate whether the incidence varies by the type of definition or tumor type.

## 2 MATERIALS AND METHODS

### 2.1 This study followed PRISMA guidelines. The protocol was not registered online

#### 2.1.1 Study eligibility

The Population, Exposure, Comparator, and Outcome (PECO) framework was used to define the eligibility criteria (Table S1). The eligibility criteria included observational cohort studies in which patients were diagnosed with malignant solid tumors and treated with ICI, evaluated for HPD by a definition that considers the pretreatment tumor growth kinetics, and in which sufficient incidence data with a numerator and denominator were provided. Additionally, non‐English‐language studies were excluded. The search strategy was reviewed according to the Peer Review of Electronic Search Strategies checklist by a clinical informationist (Appendix S1).

#### 2.1.2 Data sources

One reviewer (MK) conducted the literature search for eligible articles in the scientific and grey literature in PubMed, Embase, Web of Science, and the Cochrane Database of Systematic Reviews from database inception to June 21, 2022. Bibliographies of previous systematic reviews were additionally scanned to identify any additional eligible articles. Database search results were exported into Endnote (version 20.4), where duplicates were removed both electronically and manually.

#### 2.1.3 Study screening

To assess eligibility, pairs of two reviewers (MK and SH or YP) independently screened each title and abstract, followed by full‐text screening. Any discrepancies were discussed with a third reviewer (SH or YP) to reach a consensus.

#### 2.1.4 Risk of bias assessment

The study quality and risk of bias for studies included in the meta‐analysis were independently assessed by two authors (MK and SH) based on the Newcastle‐Ottawa Scale. A score greater than or equal to 7 was considered high‐quality. The contour‐enhanced funnel plot and the Egger test were used to assess small‐study effects. The Grading of Recommendations Assessment, Development and Evaluation (GRADE) framework was used to assess the certainty of the primary outcome (Table S2).

#### 2.1.5 Data synthesis

Data were extracted by one primary reviewer (MK) then independently reviewed by two secondary reviewers (SH and YP). The following data were extracted into an Excel spreadsheet: author, year of publication, study design, study location, study duration, HPD definition and incidence, tumor type, type of therapy, associated clinical and biological factors, and prognosis of patients. In one study^5^ which reported cohorts with urothelial carcinoma (UC) and renal cell carcinoma (RCC) arms, the larger cohort (RCC) was used to avoid correlated data. For studies with overlapping cohorts, the cohort with a longer follow‐up was included. Each study was categorized by the tumor type and the type of definition that was used in order to synthesize and review evidence of associations with incidence both quantitatively and qualitatively.

The study team made a pragmatic decision to only include studies in which HPD was defined by and evaluated in a method consistent with one of the following three seminal^6^ studies and their respective definitions—(1) RECIST‐defined progressive disease with at least a two‐fold increase in the TGR by Champiat et al^2^ (definition A), (2) at least a two‐fold increase in the RECIST‐based TGK by Saâda Bouzid et al (definition B),^1^ and (3) RECIST‐defined PD with at least a 50% increase in the TGR (ΔTGR>50) by Ferrara et al^7^ (definition C). While the definition proposed by Kato et al^3^—time‐to‐treatment failure (TTF) less than 2 months, a greater than 50% increase in tumor burden after immunotherapy, and a greater than increase in progression pace—is also recognized as one of the main definitions of HPD,^6^ the use of the immune‐related response criteria (irRC) instead of RECIST introduces three major methodological differences in HPD evaluation that complicate quantitative synthesis with the other three definitions. Firstly, Kato et al^3^ carried out evaluations for HPD only among patients with TTF <2 months instead of including all patients who were evaluable for tumor growth kinetics analysis (i.e. the availability of all three CT scans) to calculate the incidence. Secondly, a 50% increase in the tumor burden by irRC, which corresponds to the criterion for progression proposed by the Southwest Oncology Group (SWOG), corresponds to a 83.7% increase in the volume of a spherical tumor while RECIST progression corresponds to a 72.8% increase. Thirdly, irRC incorporates new lesions into the sum of the target lesions while RECIST does not.

Accordingly, studies were not eligible if (1) one of the three definitions were not used, (2) the tumor growth kinetics analysis did not specify the requirement of having three CT scans (pre‐baseline/baseline/post‐baseline), and (3) the radiological criteria used in the study incorporated new lesions into the sum of target lesions. The exclusion criteria was revised to mitigate the methodological heterogeneity arising from various operationalizations of HPD evaluation, enhancing the interpretability and clinical relevance of the current data on HPD. Authors of studies that did not meet the revised eligibility criteria were contacted to request additional information or to request an adjusted incidence according to either definition A, B, or C if possible.

The primary outcome was the HPD incidence, and meta‐analysis was performed using the generalized linear mixed model, fit using log‐odds transformation and maximum likelihood estimation based on the adaptive Gaussian hermite quadrature. The presence of statistical heterogeneity was assessed using the Cochran *Q* statistic, and the Higgins *I* ^2^ statistic was calculated to measure the degree of heterogeneity arising from true variation in the study effects. Potential sources of heterogeneity across studies were explored in univariable and multivariable meta‐regression analyses for the tumor type and the type of HPD definition, which were established a priori. Tumor types were considered as subgroups only when more than two studies were available.

For all statistical tests, two‐sided *p* values were reported, and a value less than 0.05 was considered statistically significant. As confidence intervals (CI) based on the normal approximation have been associated with poor coverage for meta‐analyses with a small number of studies or substantial heterogeneity, 95% CIs were estimated using the Student's *t*‐distribution.^8^ 95% prediction intervals were reported to quantify the extent of between‐study variation in the incidences and odds ratios. All analyses were performed using the metafor and meta packages in R version 4.2.1.

Finally, the threshold inequalities defining HPD—TGK ratio ≥ 2, TGR ratio ≥ 2, and Δ TGR > 50—were expressed such that the change in the tumor burden after immunotherapy is a function of the change in the tumor burden before immunotherapy as previously modeled by Kas et al^3^ (additional information can be found in Appendix S1). This graphical representation was used to characterize differences across definitions based on incidence data reported in studies which calculated HPD incidence for different definitions.

## 3 RESULTS

**FIGURE 1:**
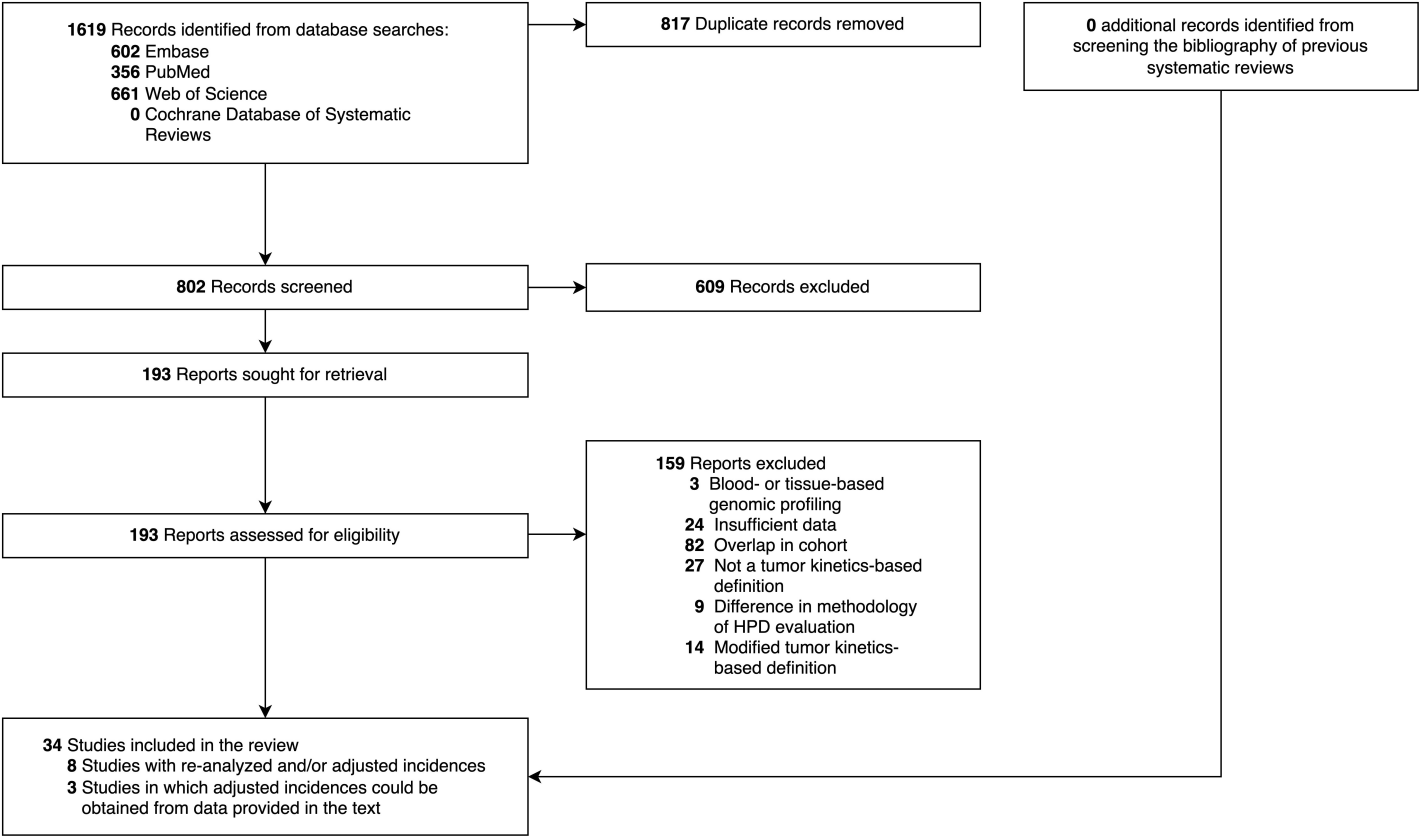
Flow diagram of study inclusion.

A total of 1619 records were identified through database searches (Figure 1). After screening and eligibility assessment, a total of three prospective and 31 retrospective observational cohort studies involving a total of 4117 patients in 12 countries were included in the systematic review and meta‐analysis (Table S3).^4^, ^5^, ^7^, ^9^, ^10^, ^11^, ^12^, ^13^, ^14^, ^15^, ^16^, ^17^, ^18^, ^19^, ^20^, ^21^, ^22^, ^23^, ^24^, ^25^, ^26^, ^27^, ^28^, ^29^, ^30^, ^31^, ^32^, ^33^, ^34^, ^35^, ^36^, ^37^, ^38^, ^39^ A list of excluded studies from the full‐text screening, with reasons for their exclusion, can be found in Table S4.

Given the broad range of operationalizations associated with HPD definitions, the studies were first categorized by the number of metrics used to estimate the tumor growth kinetics in each study. Unlike the original HPD definitions by Champiat et al,^2^ Saâda Bouzid et al,^1^ Ferrara et al,^7^ and Kato et al^3^ which proposed a single metric to describe the tumor growth kinetics, six studies^13^, ^17^, ^18^, ^20^, ^31^, ^34^ used combinations of the TGK ratio, TGR ratio, and ΔTGR to define HPD (Table S5). The studies were further categorized according to the original HPD definition that the study's definition of HPD was based on. Besides using combinations of tumor growth kinetics metrics, other operationalizations of the HPD definition included the following: (1) a change in the threshold values,^15^, ^18^, ^34^ (2) the addition^17^ or exclusion^25^ of the clinical criterion of time to treatment failure less than 2 months, and (3) the application of unidimensional RECIST^19^, ^25^, ^37^ to define a 50% change in the tumor burden, which was originally based on irRC's bidimensional measurements according to Kato et al.^3^

Studies were also categorized by the tumor type (Table S6). Patients were treated for non‐small cell lung cancer (NSCLC; *n* = 11), hepatocellular carcinoma (HCC; *n* = 5), advanced gastric cancer (AGC; *n* = 3), renal cell carcinoma (RCC; *n* = 3), head and neck squamous cell carcinoma (HNSCC; *n* = 2), melanoma (*n* = 2), sarcoma (*n* = 1), and mixed tumor types (*n* = 7). The quality of included studies was high (NOS score ≥7) in 28 (82.3%) of 34 studies, with a median score of 7.5 (IQR 7–8). Details on the quality assessment according to the Newcastle‐Ottawa Scale are reported in Table S7.

Collectively, 34 studies included in the meta‐analysis reported 558 cases of HPD among 4117 patients across seven single tumor types and mixed tumor types. The overall estimated incidence of HPD was 12.4% (95% CI, 10.2%–15.0%) and ranged from 0.0% to 36.73%, with evidence for heterogeneity that was statistically significant (*Q* = 119.32, *p* < 0.001). The moderately high degree of inconsistency (*I* ^*2*^ = 72%) demonstrated that a substantial proportion of the observed variance in the incidence reflects variance in the true effects, while the wide prediction interval (PI, 4.5%‐29.7%) indicated that the extent of dispersion in the true effects is non‐negligible. The Egger test suggested evidence of bias (*p* = 0.006), but an inspection of the contour‐enhanced funnel plot showed that the region in which the missing data were expected to be found corresponded to areas of high statistical significance—a pattern suggesting that asymmetry may not be attributed to small study effects (Figure S1).

**FIGURE 2:**
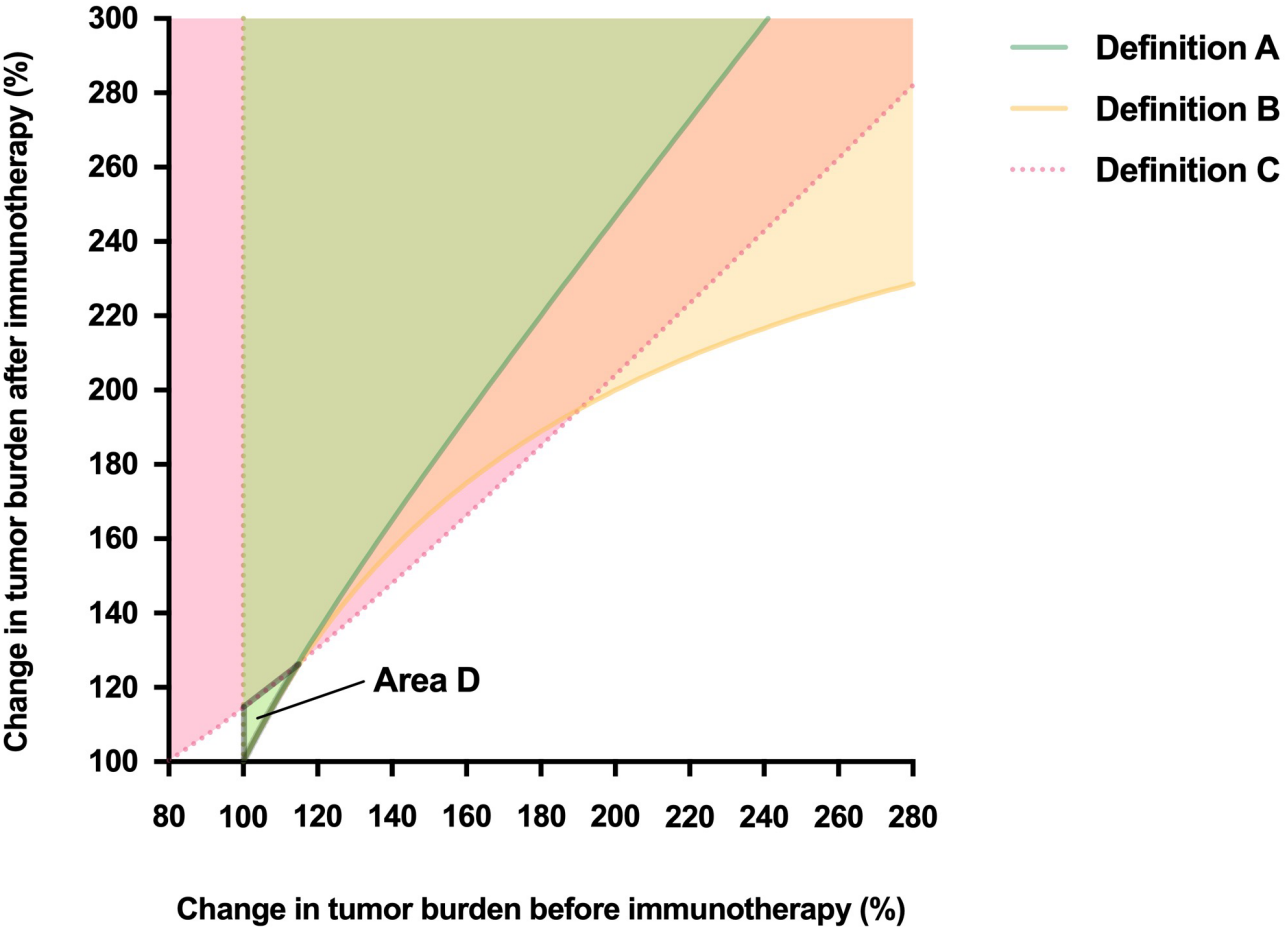
HPD incidence defined according to RECIST‐based changes in the tumor burden. Lines represent the threshold of change in the tumor growth kinetics consistent with definitions A, B, and C. Each threshold inequality graphically represents how the changes in the tumor burden before and after immunotherapy predict HPD occurrence. Area D represents a subset of patients who are evaluated as having HPD according to definition A but not definition C. The range of post‐immunotherapy tumor burden changes that characterize this area (0%–26%) suggests that these patients are more likely to meet the criteria of progression based on appearance of new lesions and not based on an increase in the tumor size that is greater than 20%. RECIST, Response Evaluation Criteria in Solid Tumors. Figure adapted from Kas et al^40^ with permission from the American Medical Association.

HPD incidence was compared across different definitions both qualitatively and quantitatively. The graphical representation of definitions A–C, based on the threshold of change in the tumor growth kinetics for each definition, revealed several qualitative differences which have not been previously described^40^ (Figure 2). First of all, as the threshold for definitions A and B are based on a ratio where both the numerator and denominator must be a value greater than zero, these two definitions can only describe HPD in patients who experience an increase in the tumor burden during both the pre‐immunotherapy and post‐immunotherapy periods. By contrast, definition C—which is based on the difference in the tumor growth rate between the two time intervals—can capture HPD patients who experience a decrease in the tumor burden during the washout‐period and an increase in the tumor burden after starting immunotherapy.

**FIGURE 3:**
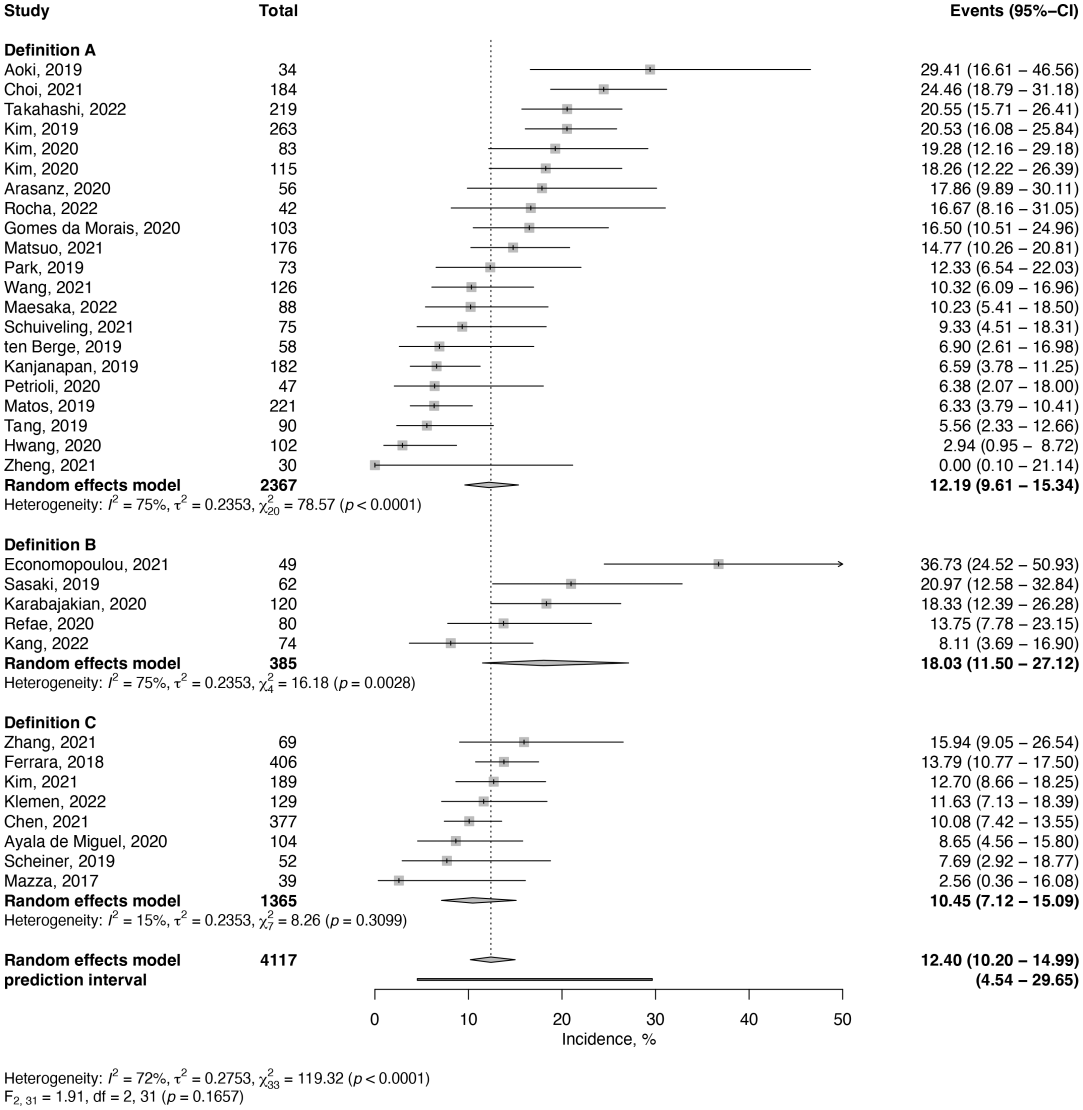
Forest plots of HPD Incidence and 95% confidence intervals (95% CI) across three HPD definitions. The 95% prediction interval (95% PI) estimates where the true effect would be found in 95% of similar studies conducted in the future.

The lowest estimated incidence of HPD was reported for definition C at 10.5% (95% CI, 7.1%–15.1%; *I* ^2^ = 15%; Figure 3), followed by definition A at 12.2% (95% CI, 9.6%–15.3%; *I* ^2^ = 75%) and definition B at 18.0% (95% CI, 11.5%–27.12%; *I* ^2^ = 75%). Univariable analysis showed that the type of definition did not have a trend for significance as a moderator for HPD incidence (*p* = 0.17).

**FIGURE 4:**
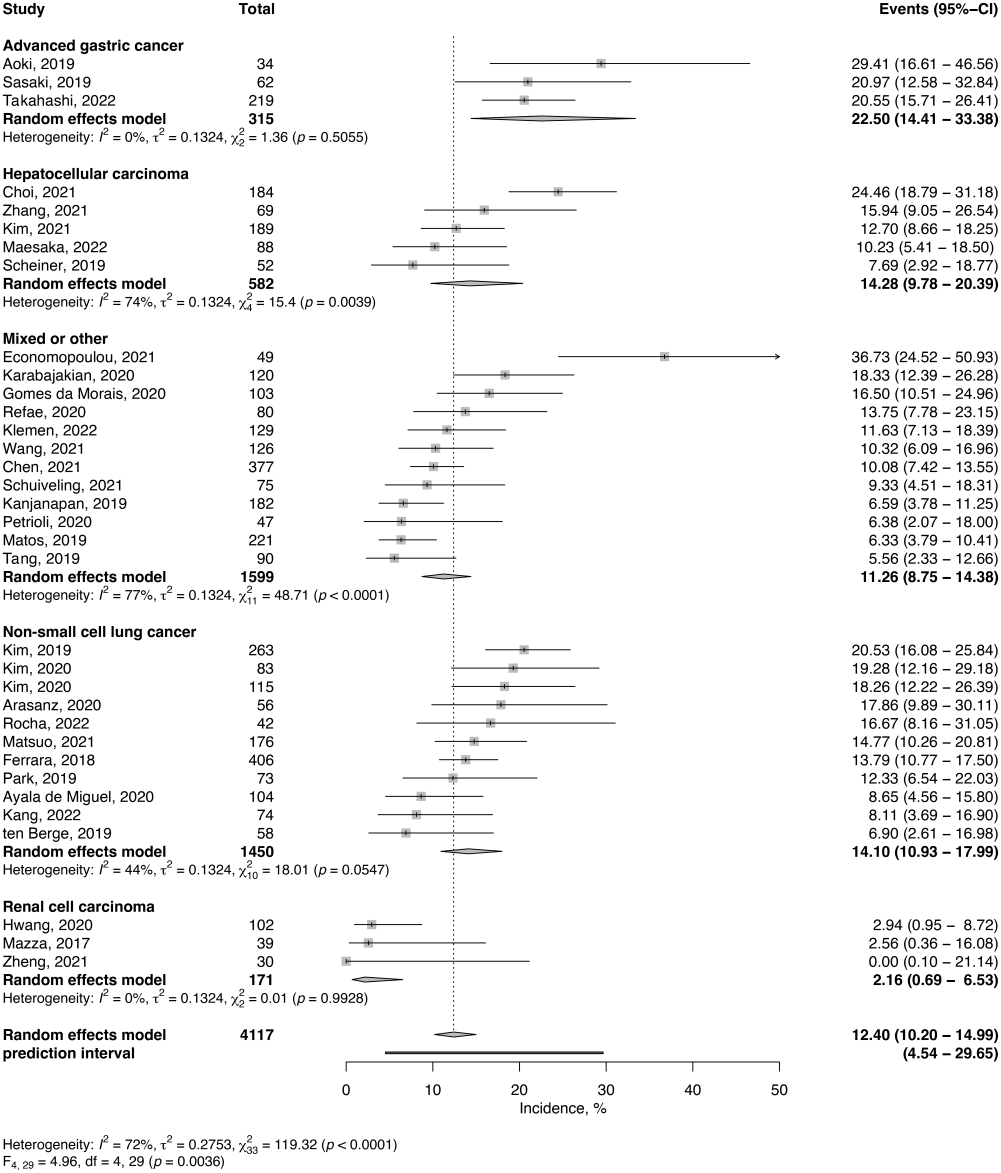
Forest plots of HPD incidence and 95% confidence intervals (95% CI) across tumor types. The 95% prediction interval (95% PI) estimates where the true effect would be found in 95% of similar studies conducted in the future.

The estimated incidence of HPD was also obtained for each tumor type subgroup (Figure 4). The estimate was highest for AGC (22.5%; 95% CI, 14.4%–33.4%; *I* ^2^ = 0%), followed by HCC (14.3%; 95% CI 9.8%–20.4%; *I* ^2^ = 74%), NSCLC (14.1%; 95% CI, 10.9%–18.0%; *I* ^2^ = 44%), and mixed or other types (11.3%; 95% CI, 8.8%–14.4%; *I* ^2^ = 77%). RCC had the lowest estimate of 2.2% (95% CI, 0.7%–6.5%; *I* ^2^ = 0%). Univariable analysis showed tumor histology as a significant moderator for HPD incidence (*p* = 0.004), with the estimated incidence of HPD significantly higher in AGC (*p* < 0.001), NSCLC (*p* = 0.002), HCC (*p* = 0.002), and mixed or other (*p* = 0.005) than in RCC.

After adjusting for the definitions used, the risk of developing HPD was higher in AGC (adjusted OR, 10.83; 95% CI, 2.14–54.65; *p* < 0.001), HCC (adjusted OR, 7.99; 95% CI, 1.68–38.13; *p* = 0.006), NSCLC (adjusted OR, 7.14; 95% CI, 1.58–32.29; *p* = 0.005), and mixed or other types (adjusted OR, 5.09; 95% CI, 1.12–23.14, *p* = 0.018) than in RCC (Table 1). After adjusting for the tumor type, Definition B (adjusted OR, 1.82; 95% CI, 1.08–3.07; *p* = 0.025) reported higher incidences of HPD than Definition C.

**TABLE 1:** Multivariable analyses results for HPD incidence.

Variable	Odds ratio (95% CI)	*p*‐Value^a^
Tumor types		
AGC	10.83 (2.14–54.65)	<0.001
HCC	7.99 (1.68–38.13)	0.006
NSCLC	7.14 (1.58–32.29)	0.005
Mixed or other	5.09 (1.12–23.14)	0.018
RCC	Reference	–
Definition types		
A (Champiat et al^2^)	1.20 (0.83–1.74)	0.335
B (Saâda‐Bouzid et al^1^)	1.82 (1.08–3.07)	0.025
C (Ferrara et al^7^)	Reference	–

^a^ Mixed‐effects analysis.

Post hoc sensitivity analyses were carried out to test the robustness of the results. Meta‐analyses were repeated after excluding studies that were prospective observational cohorts, received NOS scores fewer than 7, obtained from the grey literature, or outliers. Outliers were identified using the Baujat plot^41^ (Figure S2), in which studies found in the upper right quadrant represent studies with a substantial contribution to both the estimated heterogeneity and the pooled effect. The results were consistent with the main findings (Table S8).

Clinical and biological factors associated with HPD were not amenable to quantitative synthesis due to use of adjusted incidences in this meta‐analysis and therefore are summarized in Appendix S1.

## 4 DISCUSSION

### 4.1 Overall findings

Although differences arising from operationalizations of HPD definitions were accounted for in the meta‐analysis—as the adjusted incidence according to definition A, B, or C were obtained from the text or provided by the authors—the considerable variation observed in the literature illustrates the current challenges in conducting a proper synthesis of the evidence on HPD.

This meta‐analysis of 34 studies from 12 countries was consistent with previous findings that there is statistically significant heterogeneity in the HPD incidence.^42^ As the prediction interval estimates where the true effect would lie for 95% of similar studies, the wide prediction interval in this meta‐analysis demonstrates that the incidence varies substantially across studies; this suggests that there may be subsets of patients that are less or more likely to experience HPD.

Specifically, the odds of experiencing HPD was significantly higher for AGC, HCC, NSCLC, and mixed or other types than patients with RCC. The HPD incidence was also higher for definition B compared to C with statistical significance, which introduces uncertainty in the estimation of true HPD incidence. While our findings indicate that there may be differences in HPD incidence by the type of tumor or definition used, the evidence should be interpreted with caution due to the small number of included studies.

What further limits an interpretation of these observed differences is the fact that the mechanism behind HPD remains to be elucidated. Current hypotheses for ICI‐related HPD suggest possible association with mechanisms of adaptive resistance which involve changes in the immune contexture of the tumor microenvironment (TME).

Firstly,  an alteration of T cell subsets or the polarization of the myeloid subset in the TME have been previously reported. Marked increase in tumor‐infiltrating proliferative effector regulatory T (eT_reg_) cells was observed in tissue samples of gastric cancer patients,^43^ and a significant increase in CD4^+^CD25^+^CD127^lo^FoxP3^+^ T_reg_ cells^38^ or CD28^−^ CD4^+^ highly differentiated T‐cells (T_HD_)^39^ was independently reported from peripheral blood analysis of NSCLC patients who experienced HPD post‐immunotherapy. One study observed tumor infiltration by clusters of M2‐like epithelioid macrophages in both xenograft models and pretreatment tissue samples from NSCLC patients who experienced HPD,^44^ consistent with previous findings of dampened T‐cell‐mediated immune responses via enrichment of macrophages in the TME due to engagement between the anti‐PD‐1 Fc fragment and Fc receptors.^45^ In these studies, however, the pretreatment tumor kinetics was not considered in the assessment for HPD; as a result, patients classified as having experienced HPD may have included those with naturally aggressive disease.

Hyperprogression has also been associated with upregulation of alternative immune‐checkpoints as a compensatory mechanism. While this hypothesis was previously demonstrated with T‐cell immunoglobulin and mucin domain 3 (TIM‐3) in preclinical models,^46^ Kim et al^13^ observed the expansion of T‐cell immunoreceptor with Ig and ITIM domains (TIGIT)‐positive subtype among PD‐1^+^CD8^+^ T cells from peripheral blood.^13^

### 4.2 Implications for research

While the underlying reasons for differences across tumor types remain to be further studied, differences in the HPD incidence across tumor types may be attributable to the immune landscape of each tumor type. Based on the classification of immune subtypes from immunogenomics analysis of TCGA data,^47^ AGC's IFN‐γ dominant subtype is characterized by the lowest Th1/Th2 ratio, a high proliferation rate, and the highest intratumoral heterogeneity, while RCC's inflammatory subtype demonstrates the opposite trend: a high Th1/Th2 ratio, low proliferation rate, and the lowest intratumoral heterogeneity.

While the relationship between HPD and a tumor subtype characterized by IFN‐γ—a cytokine often associated with antitumor responses including the recruitment of CD8+ cells and upregulation of the antigen‐presenting machinery—may seem paradoxical, growing evidence shows that IFN‐γ signaling can also have varying effects depending on the balance of tumor and immune cells in the TME, each of which respond differently to IFN‐γ signaling.^48^ A recent study by Zou et al^49^ reported that patients who experienced HPD highly expressed fibroblast growth factor 2 (FGF2) and Wnt‐β‐catenin gene signatures; through preclinical models, the authors demonstrated that IFN‐γ production by CD8+ T cells may alter the PKM2/NAD^+^/β‐catenin signaling to enhance the tumor's oncogenic potential. Given the non‐negligible presence of the IFN‐γ dominant subtype in cancers with higher rates of HPD, future studies are needed to characterize how the immune landscape of each tumor type contributes to differential responses to immunotherapy, as well as how it is altered by the introduction of immunotherapeutic agents in the TME.

### 4.3 Implications for practice

After multivariable adjustment based on the tumor type, our study also found evidence that HPD incidence was higher for definition B than definition C with statistical significance. More importantly, this finding suggests that variations in the HPD incidence arising from the use of multiple definitions present challenges in understanding the phenomenon.

The consequences of using different HPD definitions are not limited to a general overestimation or underestimation of HPD incidence. As observed by the graphical representation of pretreatment and post‐treatment changes that describe patients who experience HPD (Figure 2), definition C is able to evaluate HPD even in those who experience a negative tumor growth rate in the pretreatment period. Definitions A and B, which cannot evaluate HPD in this subpopulation, may describe patients with potential differences in the clinical outcome as well.

A closer look at studies in this meta‐analysis which compared multiple definitions reveal additional concerns in current methods of HPD evaluation. While the areas of HPD incidence modeled by Kas et al^40^ suggest that definition A is more restrictive than definition C in estimating HPD incidence, two studies^14^, ^35^ from our analysis that explored multiple HPD definitions showed that incidence for the same cohort was higher for definition A than definition C. According to the graphical representation of regions defining HPD (Figure 2), this observation is possible only if a substantial portion of patients who developed HPD demonstrated changes in the pre‐ and post‐immunotherapy tumor burden that correspond to area D in Figure. This region is characterized by a post‐treatment increase in the tumor burden between 0% and 26%—a range in which the criteria for progressive disease is met not only by a quantitative change in the tumor size that is greater than 20%, but also by the appearance of new lesions. This finding suggests that HPD may have a characteristic distribution of pretreatment and post‐treatment changes in the tumor burden. At the same time, it sheds light on the current limitations of RECIST: the exclusion of new lesions in the measurement of the tumor burden may prevent a more accurate evaluation of tumor growth kinetics. These limitations may be overcome by the adoption of immune‐specific criteria that incorporate new lesions, a revision of the currently used RECIST criteria to include new lesions in the calculation of tumor burden for HPD evaluation, and ultimately by radiomics‐based approaches that directly measure the tumor volume.

### 4.4 Strengths and potential limitations

Our study has several limitations. First and foremost, this meta‐analysis consisted of observational studies, which are prone to selection bias and confounding effects. Secondly, imprecision in the estimates due to the small number of studies per subgroup and inherent clinical and statistical heterogeneity of the primary studies—constrain our interpretation of results. Thirdly, results from this meta‐analysis are based on aggregate data and not individual patient data, presenting the risk of ecological fallacy. Lastly, methodological heterogeneity (i.e., prior lines of therapy and type of immune checkpoint inhibitor) precludes an in‐depth quantitative analysis exploring clinicopathological factors with potential predictive and prognostic value. Nevertheless, our study also has strengths. All published studies including conference abstracts were screened to provide a comprehensive overview of all available data. Furthermore, additional exclusion criteria were specified at the eligibility stage in order to increase the likelihood of presenting clinically reliable estimates and interpretable data, despite inevitable methodological heterogeneity in the current data on HPD.

Evidence from this meta‐analysis supports the argument for the adoption of a uniform HPD definition. An accurate estimate of HPD incidence is necessary to characterize the potential risks associated with immunotherapy as well as provide timely diagnosis and proper care to patients who receive it. While clear biological underpinnings of HPD are currently lacking, we believe that the aggravating consequences of immunotherapy merit further attention to this phenomenon in future studies.

## AUTHOR CONTRIBUTIONS

**Min Jeong Kim:** Conceptualization (equal); data curation (lead); formal analysis (lead); investigation (lead); methodology (lead); visualization (lead); writing – original draft (lead); writing – review and editing (lead). **Seung Pyo D. Hong:** Conceptualization (supporting); investigation (supporting); methodology (supporting); writing – review and editing (supporting). **Yeonggyeong Park:** Investigation (supporting); writing – review and editing (supporting). **Young Kwang Chae:** Conceptualization (equal); methodology (supporting); supervision (lead); writing – review and editing (supporting).

## FUNDING INFORMATION

The author(s) received no specific funding for this work.

## CONFLICT OF INTEREST STATEMENT

Dr. Chae reports receiving a research grant to the institution from Abbvie, BMS, Biodesix, Lexent Bio, and Freenome; he has also received honoraria from or is in advisory boards for Roche/Genentech, BMS, AstraZeneca, Merck, Foundation Medicine, Counsyl, Neogenomics, Guardant Health, Boehringher Ingelheim, Biodesix, Immuneoncia, Lilly Oncology, Merck, Takeda, Pfizer, Tempus, Lunit, and Jazz Pharmaceuticals.

## ETHICS STATEMENT

No Institutional Review Board review or approval was necessary for the conduct of this systematic review, and consent was not required.

## Supporting information


Data S1

## Data Availability

The data used in this study can be found in the figshare database [https://figshare.com/articles/dataset/updated_extracted_data_HPD_22_08_29_xlsx/22256557].
