# Supplementary material for: Incidence of immunotherapy‐related hyperprogressive disease (HPD) across HPD definitions and cancer types in observational studies: A systematic review and meta‐analysis
Source: Cancer Med. 2024 Feb 24;13(3):e6970. doi: 10.1002/cam4.6970 (PMC10891462; doi:10.1002/cam4.6970)
Supplement: Supplementary file 1 — Data S1 [file CAM4-13-e6970-s001.docx]

**Supporting Information**

**Appendix S1.** Additional methods, database search algorithms, and qualitative synthesis of biological factors associated with HPD

**Table S1.** The PECO framework

**Table S2.** Grading of recommendations, assessment, development and evaluations (GRADE)

**Table S3.** Descriptive characteristics of of all studies included in the meta-analysis

**Table S4.** Studies excluded from full-text review, with reasons

**Table S5.** Categorization of studies included in the meta-analysis by definition

**Table S6.** Categorization of studies included in the meta-analysis by tumor type

**Table S7.** Quality of studies included in the meta-analysis according to the Newcastle-Ottawa Scale

**Table S8.** Sensitivity analyses after excluding prospective cohort studies, low-quality studies (NOS<7), or outliers

**Figure S1.** Contour-enhanced funnel plot for all studies included in the meta-analysis

**Figure S2.** Baujat plot of studies included in the meta-analysis

**Supporting Information References**

**Appendix S1**

1. Database search algorithms

| **Database** | **Search strategy** |
| --- | --- |
| PubMed | ("Immunotherapy"[mh] OR "immunotherap*"[tw] OR “immune therap*”[tw] OR “immunological therap*”[tw] OR "immunomodulat*"[tw] OR "immune checkpoint inhibitors"[mh] OR "immune checkpoint inhibitors"[tw] OR "programmed death 1"[tw] OR "pd-1"[tw] OR "programmed death ligand 1"[tw] OR "pd-l1"[tw] OR "cytotoxic t lymphocyte antigen 4"[tw] OR "ctla 4"[tw] OR "Antineoplastic Agents, Immunological" [Pharmacological Action] OR "Antineoplastic Agents, Immunological"[mh] OR "Antineoplastic Agents, Immunological" [tw] OR "Ipilimumab"[mh] OR "ipilimumab"[tw] OR “Yervoy”[tw] OR "Nivolumab"[mh] OR "nivolumab"[tw] OR “Opdivo”[tw] OR "pembrolizumab"[tw] OR “Keytruda”[tw] OR "atezolizumab"[tw] OR “Tecentriq”[tw] OR "durvalumab"[tw] OR “Imfinzi”[tw] OR "avelumab"[tw] OR “Bavencio”[tw] OR "cemiplimab"[tw] OR “Libtayo”[tw]) AND ("hyperprogress*"[tw] OR "hyper progress*"[tiab]) |
| Embase | ('immunotherapy'/exp OR 'immunotherap*':ti,ab OR 'immune therap*':ti,ab OR 'immunological therap*':ti,ab OR 'immunomodulat*':ti,ab OR 'immune checkpoint inhibitor'/exp OR 'immune checkpoint inhibitor':ti,ab OR 'antineoplastic monoclonal antibody'/exp OR 'antineoplastic monoclonal antibody':ti,ab OR 'programmed death 1':ti,ab OR 'pd-1':ti,ab OR 'programmed death ligand 1':ti,ab OR 'pd-l1':ti,ab OR 'cytotoxic t lymphocyte antigen 4'/exp OR 'ctla 4':ti,ab OR 'ipilimumab'/exp OR 'ipilimumab':ti,ab OR 'yervoy':ti,ab OR 'nivolumab'/exp OR 'nivolumab':ti,ab OR 'opdivo':ti,ab OR 'pembrolizumab'/exp OR 'pembrolizumab':ti,ab OR 'keytruda':ti,ab OR 'atezolizumab'/exp OR 'atezolizumab':ti,ab OR 'tecentriq':ti,ab OR 'durvalumab'/exp OR 'durvalumab':ti,ab OR 'imfinzi':ti,ab OR 'avelumab'/exp OR 'avelumab':ti,ab OR 'bavencio':ti,ab OR 'cemiplimab'/exp OR 'cemiplimab':ti,ab OR 'libtayo':ab,ti) AND ('hyperprogress*':ti,ab OR 'hyper progress*':ti,ab) |
| Web of Science | TS = (immunotherapy OR immunotherap* OR immune therap* OR immunological therap* OR immunomodulat* OR immune checkpoint inhibitor OR antineoplastic monoclonal antibody OR programmed death 1 OR pd-1 OR programmed death ligand 1 OR pd-l1 OR cytotoxic t lymphocyte antigen 4 OR ctla 4 OR ipilimumab OR ipilimumab OR Yervoy OR nivolumab OR Opdivo OR pembrolizumab OR Keytruda OR atezolizumab OR Tecentriq OR durvalumab OR Imfinzi OR avelumab OR avelumab OR Bavencio OR cemiplimab OR Libtayo) AND TS = (hyperprogress* OR hyper progress*) |
| Cochrane Database of Systematic Reviews | (immunotherapy OR immunotherap* OR immune therap* OR immunological therap* OR immunomodulat* OR immune checkpoint inhibitor OR antineoplastic monoclonal antibody OR programmed death 1 OR pd-1 OR programmed death ligand 1 OR pd-l1 OR cytotoxic t lymphocyte antigen 4 OR ctla 4 OR ipilimumab OR Yervoy OR nivolumab OR Opdivo OR pembrolizumab OR Keytruda OR atezolizumab OR Tecentriq OR durvalumab OR Imfinzi OR avelumab OR avelumab OR Bavencio OR cemiplimab OR Libtayo) AND (hyperprogress* OR hyper progress*) |

2. Additional Methods

The threshold equations to predict HPD based on RECIST changes in the tumor burden before and after immunotherapy, as previously modeled by Kas et al^1^, were derived as follows:

1. **Saâda Bouzid et al (definition B)^2^**

The authors described the tumor growth kinetics (TGK) as the change in the sum of the diameters (based on RECIST, and represented here as D) per unit time:’


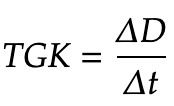


(1)

HPD was defined as a change in the TGK ratio that is greater than or equal to 2, such that
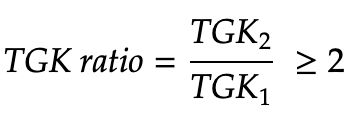


Substituting (1),


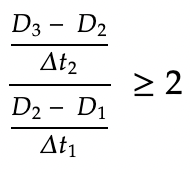


where D_1_, D_2,_ and D_3_ represent the tumor size measured at the pre-baseline, baseline, and post-baseline CT imaging respectively; Δt_1_ represents the time interval between the pre-baseline and baseline CT scans, and Δt_2_ represents the time interval between the baseline and post-baseline CT scans.

Based on the ideal assumption that the time interval between each consecutive CT scan is

identical (Δ*t_1_* = Δ*t_2_*),

| 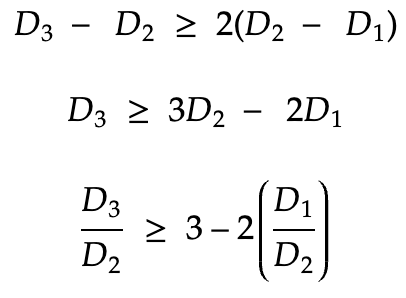  (2) | (2) |
| --- | --- |

Using *y* to represent the change in the tumor size between the post-baseline and baseline imaging (*D_3_* /*D_2_*) and *x* to represent the change between the post-baseline and baseline imaging (*D_2_* /*D_1_*), (2) can be rewritten as


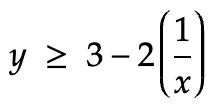


1. **Champiat et al (definition A)^3^**

The following assumptions hold true in defining the tumor growth rate (TGR), according to the authors:

- 1. The volume of the tumor burden is approximated as a sphere, such that


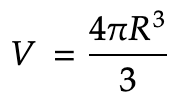


Since the radius of a sphere is equivalent to half of the sum of diameters,
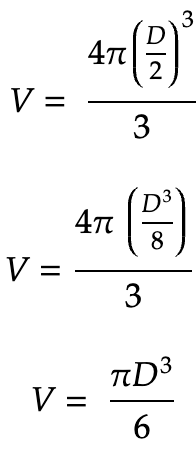


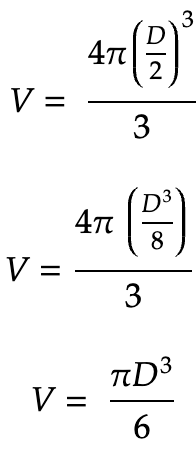


(3)

- 1. The dynamics of tumor growth is described by the exponential growth model, where


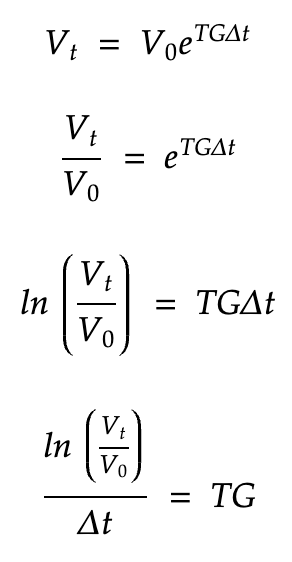


(4)

Substituting (3),


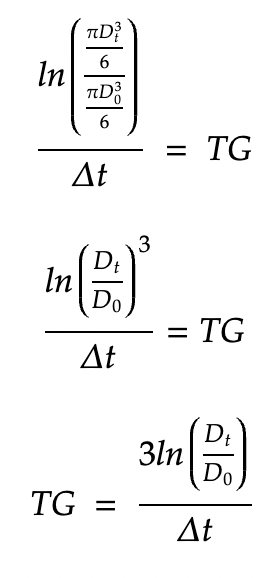


(5)

- 1. TGR is defined as the percentage change in the tumor volume per month:


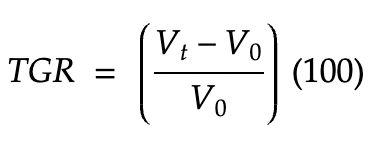


Substituting (4),


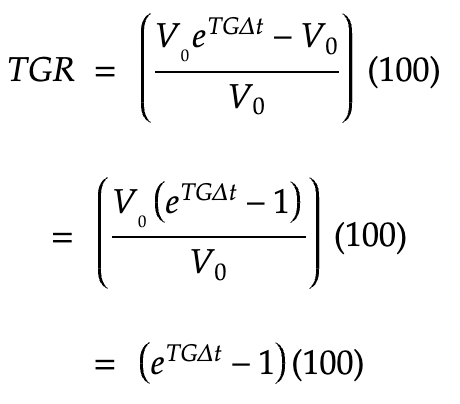


(6)

Champiat et al^3^ defined HPD as RECIST-defined progressive disease (PD) and TGR greater than or equal to 2, or


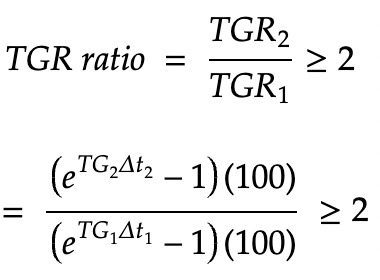


Substituting (5),


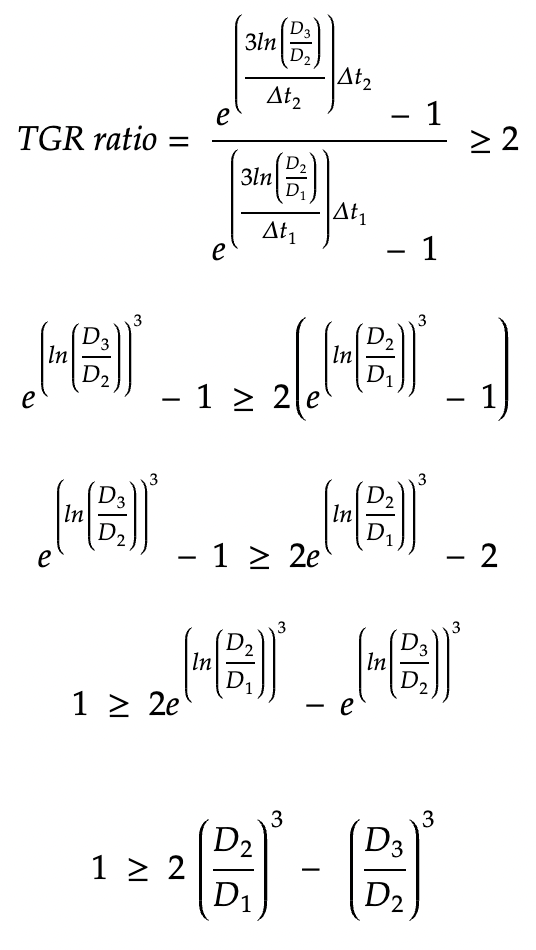


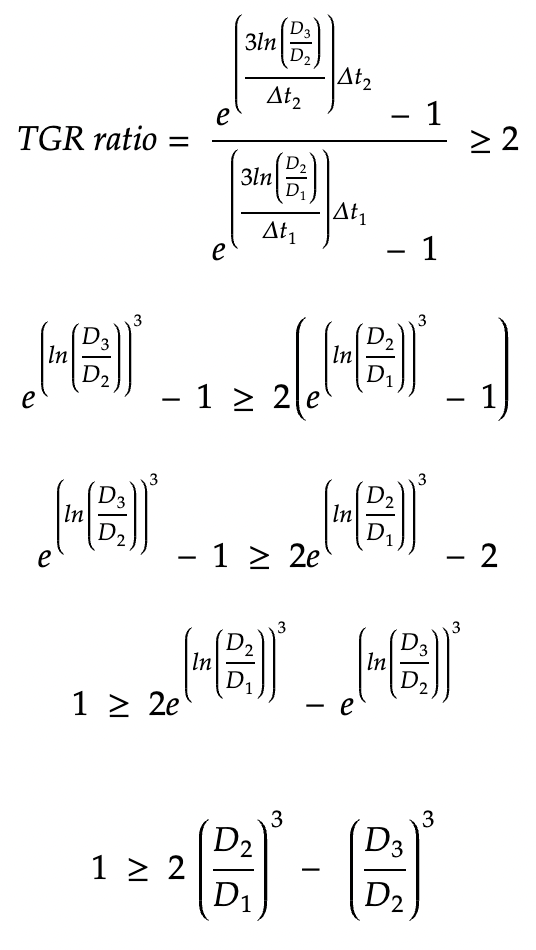


(7)

Using *y* to represent the change in the tumor size between the post-baseline and baseline imaging (*D_3_* /*D_2_*) and *x* to represent the change between the post-baseline and baseline imaging (*D_2_* /*D_1_*), (7) can be rewritten as


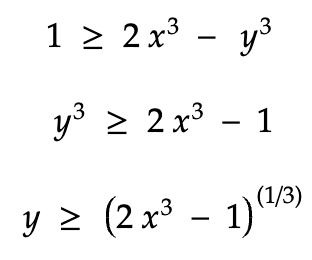


1. **Ferrara et al (definition C)^4^**

The authors defined HPD as RECIST-defined progressive disease (PD) and a change in the tumor growth rate that is greater than 50%, or


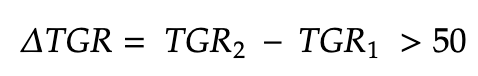


Substituting (6),


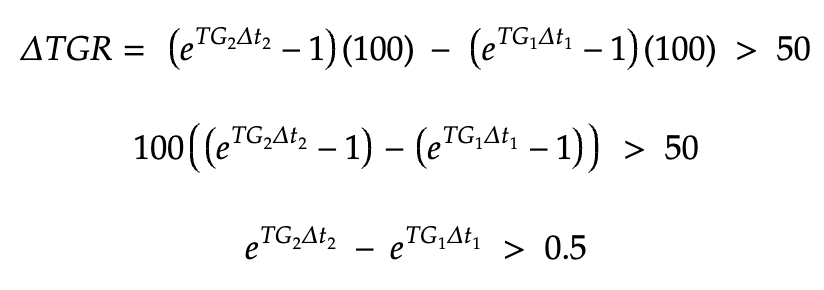


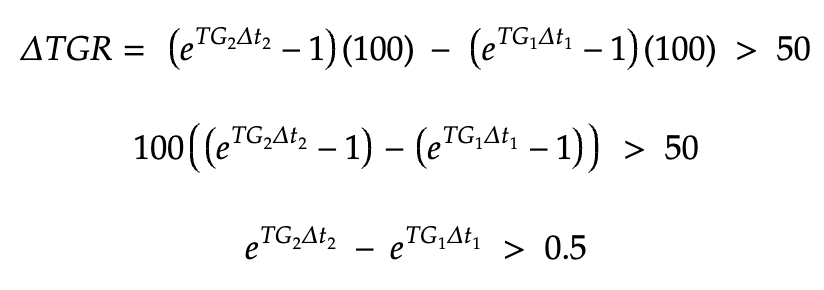


Substituting (5),


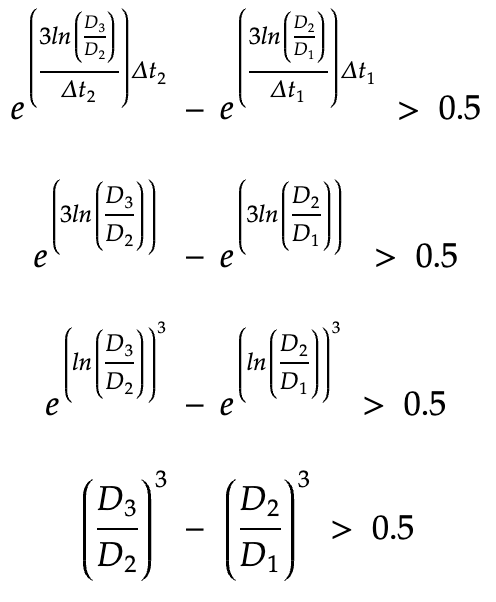


(8)

Using *y* to represent the change in the tumor size between the post-baseline and baseline imaging (*D_3_* /*D_2_*) and *x* to represent the change between the post-baseline and baseline imaging (*D_2_* /*D_1_*), (8) can be rewritten as


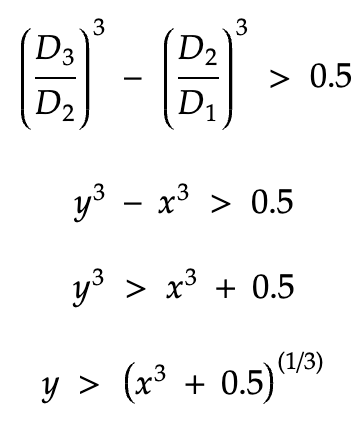


3. Qualitative synthesis of biological factors associated with HPD.

Based on a qualitative review, a higher number of metastatic sites,^4–8^ liver metastasis,^5–7,9^ an elevated neutrophil-to-lymphocyte ratio (NLR)^10–12,13^ and higher levels of baseline lactate dehydrogenase (LDH) levels^7,9,11^ were factors which were most frequently reported to have a significant association with HPD. Opposite associations were shown for age^6,14,15^ and therapeutic strategy^5,16^ (monotherapy versus combination therapy). Genomic associations^9,14,17^ were not validated in more than one study. Out of thirteen studies for which comparisons for prognosis were available between hyperprogressors and non-HPD progressors, no observable differences were found among tumor types except for advanced NSCLC, for which all five cohorts^4,18–21^ showed significant differences in the outcome (Table S4).

**Table S1.** The PECO Framework

| Population | Patients diagnosed with malignant solid tumors |
| --- | --- |
| Exposure | Treatment with immune checkpoint inhibitors |
| Comparator | No HPD present upon the first post-treatment evaluation |
| Outcome | Incidence of HPD, where HPD was defined by integrating the pretreatment tumor growth kinetics |

**Table S2.** Grading of recommendations, assessment, development and evaluations (GRADE)

| Outcome | No. of studies | Study design | Certainty assessment | | | | | Effect | | Certainty |
| --- | --- | --- | --- | --- | --- | --- | --- | --- | --- | --- |
|  |  |  | Risk of bias | Imprecision | Inconsistency | Indirectness | Publication Bias | No of patients | Incidence (95% CI) |  |
| HPD incidence by tumor type | | | | | | | | | | |
| Subgroup: advanced gastric cancer | 3 | observational study | not serious | serious | Low | Low | Moderate | 315 | 22.50 (14.41 − 33.38) | ⊕◯◯◯ Very low |
| Subgroup: hepatocellular carcinoma | 5 | observational study | not serious | not serious | Moderate | Low |  | 582 | 14.28 (9.78 − 20.39) | ⊕⊕◯◯ Low |
| Subgroup: mixed or other cancer types | 12 | observational study | not serious | not serious | High | Low |  | 1599 | 11.26 (8.75 − 14.38) | ⊕◯◯◯ Very low |
| Subgroup:  non-small cell lung cancer | 11 | observational study | not serious | not serious | Low | Low |  | 1450 | 14.10 (10.93 − 17.99) | ⊕⊕◯◯ Low |
| Subgroup:  renal cell carcinoma | 3 | observational study | not serious | serious | Low | Low |  | 171 | 2.16 (0.69 − 6.53) | ⊕◯◯◯ Very low |
| HPD incidence by the type of HPD definition used | | | | | | | | | | |
| Subgroup: definition A | 21 | observational study | not serious | not serious | High | Low | Moderate | 2367 | 12.19 (9.61 − 15.34) | ⊕◯◯◯ Very low |
| Subgroup: definition B | 5 | observational study | not serious | not serious | High | Low |  | 385 | 18.03 (11.50 − 27.12) | ⊕◯◯◯ Very low |
| Subgroup: definition C | 8 | observational study | not serious | not serious | Low | Low |  | 1365 | 10.45 (7.12 − 15.09) | ⊕⊕◯◯ Low |

**Table S3.** Descriptive characteristics of all studies included in the meta-analysis

| **Source** | **Study design** | **Duration** | **Tumor type** | **Treatment types** | **HPD criteria** | | **HPD incidence (%)** | **Clinical and biological factors associated with HPD** | **Prognostic outcome of HPD** | |
| --- | --- | --- | --- | --- | --- | --- | --- | --- | --- | --- |
|  |  |  |  |  |  |  |  |  | HPD vs non-HPD | HPD vs PD without HPD |
| Mazza et al,^22^ 2017^a^ (France) | single-center retrospective study (clinical trial data) | February 2016 to July 2016 | RCC | PD-1 inhibitor monotherapy | RECIST-defined PD and ΔTGR > 50 | | 1/39 (2.5) | NA | NA | NA |
| Ferrara et al,^4^ 2018 (France) | multicenter retrospective study | August 2011 to April 2017 | NSCLC | PD-1/PD-L1 inhibitor monotherapy or in combination (with immunotherapy, anti-EGFR, or chemotherapy) | RECIST-defined PD and ΔTGR > 50 | | 56/406 (13.8) | higher number of metastatic sites (>2; *P*=.006) | NA | Median OS: 3.4 vs. 6.2 mo (HR, 2.18; *P*=.003) |
| Aoki et al,^23^ 2019 (Japan) | single-center retrospective study; | June 2009 to September 2018 | AGC | PD-1 inhibitor monotherapy or in combination with chemotherapy | RECIST-defined PD and TGR ratio ≥ 2 (on-treatment vs. pre-treatment) | | 10/34 (29.4) | no significant associations | OS: HR, 4.7 (*P*=.002)  PFS: HR, 3.4 (*P*=.004) | OS: HR, 2.1 (*P*=.17)  PFS: HR, 1.1 (*P*=.76) |
| Kanjanapan et al,^24^ 2019 (Canada) | single-center retrospective study (clinical trial data) | August 2012 to September 2016 | mixed | PD-1/PD-L1 inhibitor monotherapy or in combination (with costimulatory molecule, microenvironment modulator, CTLA-4 inhibitor, or other checkpoint inhibitor) | RECIST-defined PD and TGR ratio ≥ 2 (on-treatment vs. pre-treatment) | | 12/182 (6.6) | female gender (*P*=.01) | Median OS: 5.9 vs 14.3 mo (HR, 1.7; *P*=.11)  Median PFS: 1.6 vs 2.8 mo (HR, 3.7; *P*<.001) | NA |
| Scheiner et al,^25^ 2019 (Austria and Germany) | multicenter retrospective study | July 10, 2015 to December 31, 2018 | HCC | PD-1 inhibitor monotherapy | RECIST-defined PD and ΔTGR > 50 | | 4/52 (7.7) | NA | NA | NA |
| Tang et al,^26^ 2019^a^ (China) | single-center retrospective study (two clinical trials data) | April 2016 to September 2018 to December 2016 to September 2018 | melano-ma | PD-1 inhibitor monotherapy | RECIST-defined PD and TGR ratio ≥ 2 (on-treatment vs. pre-treatment) | | 5/90 (5.6) | NA | NA | NA |
| Sasaki et al,^9^ 2019^b^ (Japan) | single-center retrospective study | September 2017 to April 2018 | AGC | PD-1 inhibitor monotherapy | definition 1: RECIST-defined PD, tumor burden increase >50%, and TGK ratio ≥ 2 (on-treatment vs. pre-treatment) | | 13/62 (21.0) | ECOG PS (1 or 2 vs 0, *P*=.003), liver metastases (*P*=.029), sum of target lesion diameters at baseline (median; 104.2 mm vs 44.9 mm; *P*=.003), baseline ANC (median; 4,490/μl vs 2,720/μl, *P=*.002), CRP level (median; 4.0 mg/dl vs 0.50 mg/dl; *P=*.006), LDH level (median; 396.0 U/l vs. 179.5 U/l; *P=*.006), ANC levels 4 weeks after treatment initiation (median; 7740/μl vs 4490/μl; *P*=.002), CRP levels 4 weeks after treatment initiation (median; 8.3 mg/dl vs 4.0 mg/dl), *KRAS* amplification (*P*=.018), *FBXW7* mutation (*P*=.018) | Median OS: HR, 9.16 (2.3 mo vs not reached; *P*<.001)  Median PFS: HR, 4.82 (0.7 vs 2.4 mo; *P*<.001) | NA |
|  |  |  |  |  | definition 2^d^: RECIST-based TGK ratio ≥ 2 (on-treatment vs. pre-treatment) | | 13/62 (21.0) | NA | NA | NA |
| Kim et al,^18^ 2019 (Korea) | single-center retrospective study | April 2014 to November 2018 | NSCLC | PD-1/PD-L1 inhibitor monotherapy | definition 1: RECIST-defined PD, TGK ratio and TGR ratio ≥ 2 (on-treatment vs. pre-treatment) | | 54/263 (20.5) | a lower frequency of effector/memory subtype (CCR7⁻CD45RA⁻) in total CD8⁺ T cells (*P*<.001) in peripheral blood, a higher frequency of severely exhausted CD8⁺ T cells (TIGIT⁺) in total tumor-reactive (PD-1⁺) CD8⁺ T cells (P<.001) in peripheral blood | Median OS and median PFS: significantly shorter in patients with HPD | Median OS: 1.6 vs 6.7 mo (HR, 5.1; 95% CI, 3.138–8.226; overall log-rank *P*<.001)  Median PFS: 0.6 vs 1.6 mo (HR, 4.6; 95% CI 2.868–7.440; overall log-rank *P*<.001) |
|  |  |  |  |  | definition 2^d^: RECIST-defined PD and TGR ratio ≥ 2 (on-treatment vs. pre-treatment) | | 54/263 (20.5) | NA | NA | NA |
|  |  |  |  |  | definition 3: RECIST-defined PD and TGK ratio ≥ 2 (on-treatment vs. pre-treatment) | | 55/263 (20.9) |  |  |  |
|  |  |  |  |  | definition 4: TTF < 2 mo | | 98/263 (37.3) |  |  |  |
| ten Berge et al,^27^ 2019 (Netherlands) | single-center retrospective study | June 2015 to September 2017 | NSCLC | PD-1 inhibitor monotherapy | RECIST-defined PD and TGR ratio ≥ 2 (on-treatment vs. pre-treatment) | | 4/58 (6.9) | no significant correlations | OS: 2.3 vs 12.3 mo (*P*=.04) | NA |
| Park et al,^19^ 2019^a^ (Korea) | single-center retrospective study | June 2016 to October 2018 | NSCLC | PD-1/PD-L1 inhibitor monotherapy | definition 1^d^: RECIST-defined PD and TGR ratio ≥ 2 (on-treatment vs. pre-treatment) | | 9/73 (12.3) | no significant correlations | NA | Median OS (same for definition 1 and 2): 1.6 vs 2.1 mo (*P*<.001)  Median PFS (same for definition 1 and 2): 2.4 vs 5.2 mo (*P*=.002) |
|  |  |  |  |  | definition 2: RECIST-defined PD and TGK ratio ≥ 2 (on-treatment vs. pre-treatment) | | 11/73 (15.1) |  |  |  |
|  |  |  |  |  | definition 3: RECIST-defined PD and ΔTGR > 50 | | 0/73 (0.0) |  |  | NA |
| Petrioli et al,^28^ 2020 (Italy) | single-center retrospective study (prospectively collected registry data) | January 2016 to December 2018 | mixed | PD-1 inhibitor monotherapy | RECIST-defined PD and TGR ratio ≥ 2 (on-treatment vs. pre-treatment) | | 3/47 (6.4) | NA | NA | NA |
| Arasanz et al,^20^ 2020 (Spain) | single-center prospective study | September 2017 to May 2019 | NSCLC | PD-1/PD-L1 inhibitor monotherapy | RECIST-defined PD and TGR ratio ≥ 2 (on-treatment vs. pre-treatment) | | 10/56 (17.9) | smoking (*P=*.035), change in the percentage of highly differentiated (CD28⁻) CD4⁺ T cells during the first cycle of nivolumab therapy > 1.3 (*P*=.008) | Median OS: 3.5 vs 12.6 mo (*P*=.006)  Median PFS: 1.4 vs 2.5 mo (*P*<.001) | Median PFS: significantly shorter in patients with HPD (*P*=.04) |
| Refae et al,^14^ 2020 (France) | single-center retrospective study | April 2018 to August 2018 | mixed | PD-1/PD-L1 inhibitor monotherapy | RECIST-based ratio ≥ 2 (on-treatment vs. pre-treatment) | | 11/80 (13.8) | univariate: immune-related toxicity (*P*=.016)  multivariate: older age (≥ 70 years; *P*=.006), *VEGFR2* rs1870377 T/A or A/A (*P*=.007), *PDL1* rs2282055 T/G or G/G (*P*=.01) | OS: HR, 6.1 (95% CI, 1.58-23.45; overall log-rank *P*=.003) | NA |
| Hwang et al,^6^ 2020^b^ (Korea) | single-center retrospective study | February 2015 to June 2018 | RCC | PD-1/PD-L1 inhibitor monotherapy or in combination (with targeted therapy, VEGF inhibitor, or tyrosine kinase inhibitor) | definition 1: TTF < 2 mo, RECIST-defined PD, tumor burden increase > 50%, and TGR ratio ≥ 2 (on-treatment vs. pre-treatment) or extensive (≥10) new measurable lesions regardless of changes in TGR | | 1/102 (1.0) | univariate: older age (≥ 65 years; *P*=.02), liver metastasis (*P*=.009), higher number of metastatic sites (>2; *P*=.02)  multivariate: creatinine > 1.3 mg/dL (*P*=.03), increase in lymphocyte count > 30% (negative association; *P*=.01), tumor type (UCC vs RCC; *P*=.02) | NA | NA |
|  |  |  |  |  | definition 2^d^: RECIST-defined PD and TGR ratio ≥ 2 | | 3/102 (2.9) | NA |  |  |
| Matos et al,^5^ 2020 (Spain) | single-center retrospective study (clinical trial data) | January 2012 to June 2018 | mixed | PD-1/PD-L1 inhibitor monotherapy or in combination (with other immunotherapeutic agents) | definition 1: RECIST-defined PD in the first 8 weeks after treatment initiation and minimum increase in the measurable lesions at least 10 mm in size plus: (i) > 40% increase in the sum of target lesions (on treatment vs. baseline) and/or (ii) > 20% increase in the sum of target lesions with the appearance of new lesions in at least two different organs (on treatment vs. baseline) | | 29/270 (10.7) | no significant correlations | NA | Median OS: 5.2 vs 7.3 mo (HR =1.73; 95% CI, 1.05–2.85; *P*=0.04) |
|  |  |  |  |  | definition 2^d^: RECIST-defined PD and TGR ratio ≥ 2 | | 14/221 (6.3) | liver metastasis (*P*=.001), higher number of metastatic sites (>2; *P*=.038), lower TGR value in the reference period (*P*<.001), higher TGR value in the experimental period (*P*=.002), type of therapy (combination vs monotherapy; *P*=.019) |  | Median OS: 4.2 vs. 6.7 mo (HR 1.4; 95% IC, 0.70–2.77; *P*=.346) |
| Karabajakian et al,^12^ 2020 (France) | single-center retrospective study | March 2014 to November 2018 | HNSCC | PD-1/PD-L1 inhibitor monotherapy or in combination (with CTLA-4 inhibitor or KIR inhibitors) | RECIST-based ratio ≥ 2 (on-treatment vs. pre-treatment) | | 22/120 (18.3) | higher NLR (> 5; *P*<.04) | Median OS: 3.8 vs. 14.6 mo (HR, 2.2; 95% CI, 1.1 - 4.3; *P*=.002)  Median PFS: 1.9 vs. 3.9 mo (HR, 2.8; 95% CI, 1.4 to 5.6; *P*<.001) | NA |
| Ayala de Miguel,^8^ 2020^a^ (Spain) | single-center retrospective study | December 2015 to December 2019. | NSCLC | PD-1/PD-L1 inhibitor monotherapy | RECIST-defined PD and ΔTGR > 50% | | 9/104 (8·65) | higher number of metastatic sites (>2; *P*=.026) | OS: 12.6 vs 2.6 mo (HR, 13.9; *P*<.001) | NA |
| Kim et al^21^, 2020 (Korea) | single-center retrospective study | July 2016 to June 2018 | NSCLC | PD-1 inhibitor monotherapy | RECIST-defined PD and TGR ratio ≥ 2 (on-treatment vs. pre-treatment) | | 16/83 (19.3) | higher frequency of increased pleural/pericardial effusion in patients with preexisting pleural or pericardial metastasis compared to non-HPD progressors after one cycle of nivolumab (*P*=.005); [after one/two doses of nivolumab] decrease in circulating albumin (*P*=.030), increases in white blood cell count (WBC) (*P*=.005), aspartate aminotransferase (AST) (*P*=.023), and CRP level (*P*=.046) | NA | Median OS: 2.2 vs. 4.1 mo (HR, 1.18; 95% CI, 0.59–2.35; *P*=.641)  Median PFS: 0.43 vs. 1.35 mo (HR, 3.654; 95% CI, 1.667–8.010; *P*=.001) |
| Gomes da Morais et al,^29^ 2020^b^ (Spain) | single-center retrospective study (clinical trials data) | January 2017 to December 2018 | others | PD-1/PD-L1 inhibitor monotherapy or dual therapy, other immunotherapies | definition 1: RECIST-defined PD and TGK ratio ≥ 2 (on-treatment vs. pre-treatment) | | 17/103 (16.5) | NA | Median PFS: 1.1 vs. 1.6 mo (95% CI, 0.73–1.60 mo; *P*=.01) | NA |
|  |  |  |  |  | definition 2^d^: RECIST-defined PD and TGR ratio ≥ 2 (on-treatment vs. pre-treatment) | | 17/103 (16.5) |  | Median PFS: 1.4 vs. 1.6 mo (95% CI, 1.05 –1.75 mo; *P*=.04) |  |
|  |  |  |  |  | definition 3: RECIST-defined PD and ΔTGR > 100 | | 6/103 (5.8) |  | Median PFS: 0.6 vs. 1.6 mo (95% CI, 0.00–1.59 mo; *P*=.01) |  |
|  |  |  |  |  | definition 4: RECIST-defined PD, ≥ 10 mm increase in measurable lesions, tumor burden increase > 40%, and/or tumor burden increase > 20% with new lesions in ≥ 2 different organs | | 23/103 (22.3) |  | Median PFS: 1.4 mo (95% CI, 1.08–1.71 mo; *P*=.01) |  |
| Kim et al,^30^ 2020^b^ (Korea) | single-center retrospective study | April 2015 to April 2019 | NSCLC | PD-1/PD-L1 inhibitor monotherapy | definition 1: RECIST-defined PD, TGK ratio and TGR ratio ≥ 2 (on-treatment vs. pre-treatment) | | 19/115 (16.5) | higher frequency of CD39⁺ subtype in total CD8⁺ T cells in peripheral blood when compared to non-hyperprogressive progressors (*P*<.05) | NA | NA |
|  |  |  |  |  | definition 2^d^: RECIST-defined PD and TGR ratio ≥ 2 (on-treatment vs. pre-treatment) | | 21/115 (18.26) | NA | NA | NA |
| Zhang et al,^31^ 2021^b^ (China) | single-center retrospective study | March 2017 to January 2020 | HCC | PD-1 inhibitor monotherapy | definition 1: TTF < 2 mo, RECIST-defined PD, ΔTGR > 50%, and TGR ratio ≥ 2 (on-treatment vs. pre-treatment) | | 10/69 (14.5) | multivariate: hemoglobin level (*P=*.01), PVTT (*P*=.03), and Child-Pugh score (*P*=.008) | Median OS: HR, 4.79; 95% CI, 2.18-10.485; *P*<.001 | Median OS: HR, 2.50; 95% CI 1.11-5.67; *P*=.05 |
|  |  |  |  |  | definition 2^d^: RECIST-defined PD and ΔTGR > 50 | | 11/69 (15.9) | NA | NA | NA |
| Rocha et al,^32^ 2021 (Spain) | single-center retrospective study | October 2015 to September 2017 | NSCLC | PD-1 inhibitor monotherapy | definition 1^d^: RECIST-defined PD and TGR ratio ≥ 2 (on-treatment vs. pre-treatment) | | 7/42 (16.7) | NA | Median OS (definition 1): 5.0 mo and 6.7 mo (*P*=.19) | NA |
|  |  |  |  |  | definition 2: RECIST-defined PD and TGK ratio ≥ 2 (on-treatment vs. pre-treatment) | | 7/42 (16.7) |  |  |  |
|  |  |  |  |  | definition 3: RECIST-defined PD, ≥ 10 mm increase in measurable lesions, tumor burden increase > 40%, and/or tumor burden increase > 20% with new lesions in ≥ 2 different organs | | 6/42 (14.3) |  |  |  |
| Economopoulou et al,^15^ 2021^c^ (Greece) | single-center retrospective study | October 2015 to July 2020 | HNSCC | PD-1/PD-L1 inhibitor monotherapy or in combination with CTLA-4 inhibitor | RECIST or iRECIST-based ratio ≥ 2 (on-treatment vs. pre-treatment) | | 18/49 (36.7) | univariate: younger age (median 54 vs 65; *P*=.031)  multivariate: primary site (oral cavity vs others; *P*=.028), line of ICI (2nd or 3rd vs. 1st; *P*=.012) | Median OS: 6.53 vs. 15 mo (P=.002)  Median PFS: 1.8 vs. 6.1 mo (*P*<.001) | NA |
| Kim et al,^10^ 2021^b^ (Korea) | multicenter retrospective study | March 2016 to June 2019 | HCC | PD-1 inhibitor monotherapy | definition 1: RECIST-defined PD, TGK ratio and TGR ratio ≥ 4 (on-treatment vs. pre-treatment), and ΔTGR > 40 | | 24/189 (12.7) | higher NLR (>4.125; *P*<.001) | NA | Median OS: 59 vs. 96 days (HR, 2.238; 95% CI, 1.233–4.062; overall log-rank *P*<.001)  Median PFS: 23 vs. 48 days (HR, 2.194; 95% CI, 1.214–3.964; overall log-rank P<.001) |
|  |  |  |  |  | definition 2^d^: RECIST-defined PD and ΔTGR >50 | |  | NA |  | NA |
| Zheng et al,^33^ 2021 (China) | single-center retrospective study | June 2016 to October 2019 | RCC (clear cell) | PD-1 inhibitor monotherapy | RECIST or iRECIST-defined PD and TGR ratio ≥ 2 (on-treatment vs. pre-treatment) | | 0/30 (0.0) | NA | NA | NA |
| Schuiveling et al,^34^ 2021^c^ (Netherlands) | single-center retrospective study | January 2013 to March 2019 | melano-ma | PD-1 inhibitor monotherapy or in combination with CTLA-4 inhibitor, CTLA-4 inhibitor monotherapy | definition 1: TTF < 2 mo, RECIST-defined tumor burden increase > 50%, and TGR ratio ≥ 2 (on-treatment vs. pre-treatment) | | 1/75 (1.3) | NA | NA | NA |
|  |  |  |  |  | definition 2^d^: RECIST-defined PD and TGR ratio ≥ 2 (on-treatment vs. pre-treatment) | | 7/75 (9.3) |  |  |  |
| Chen et al,^7^ 2021 (China) | single-center retrospective study | January 2015 to January 2019 | mixed | PD-1/PD-L1 inhibitor monotherapy or dual therapy, or in combination (with chemotherapy/targeted therapy) | RECIST-defined PD and ΔTGR > 50% | | 38/377 (10.1) | higher number of metastatic sites (>2; *P*<.001), ECOG PS ≥ 2, liver metastasis (*P*<.001), higher LDH level (>ULN; *P*=.007), tumor type (*P*<.01), *KRAS* mutation (in colorectal cancer patients; *P*=.039), increase in tumor markers within one month of therapy for lung squamous carcinoma (*P*=.022), lung adenocarcinoma (*P*<.01), colorectal cancer (*P*<.01), pancreatic cancer (*P*<.01), and cholangiocarcinoma (*P*=.034) | NA | Median OS: 3.6 vs. 7.3 mo (*P*<.01) |
| Matsuo et al,^16^ 2021 (Japan) | single-center retrospective study | February 2016 to March 2020 | NSCLC | PD-1/PD-L1 inhibitor monotherapy or in combination with chemotherapy | RECIST-defined PD and TGR ratio ≥ 2 (on-treatment vs. pre-treatment) | | 26/176 (14.8) | type of therapy (monotherapy vs combination therapy; *P*=.031) | NA | NA |
| Choi et al,^13^ 2021^b^ (Korea) | single-center retrospective study | July 2017 to June 2020 | HCC | PD-1 inhibitor monotherapy | definition 1: RECIST-defined PD and TGK ratio and TGR ratio ≥ 4 (on-treatment vs. pre-treatment) | | 18/194 (9.3) | Higher ∆NLR at 4 weeks after treatment initiation (*P*<.001) | NA | Median OS: HR, 2.25 (95% CI, 1.31-3.85; overall log-rank P<.001) |
|  |  |  |  |  | definition 2^d^: RECIST-defined PD and TGR ratio ≥ 2 (on-treatment vs. pre-treatment) | | 45/184 (24.5) | NA | NA | NA |
| Wang et al,^17^ 2021 (China) | single-center retrospective study | February 2016 to January 2020 | mixed (gastro-intestin-al) | PD-1/PD-L1 inhibitor monotherapy | new lesions not included in SLD | definition 1^d^: RECIST-defined PD and TGR ratio ≥ 2 (on-treatment vs. pre-treatment) | 6/126 (4.8) | NA | NA | Median OS: HR, 2.62; 95% CI, 0.99– 6.96; *P*=.045 |
|  |  |  |  |  |  | definition 2: RECIST-defined PD and TGK ratio ≥ 2 (on-treatment vs. pre-treatment) | 7/126 (5.6) |  |  | Median OS: HR, 1.45; 95% CI, 0.56– 3.73; *P*=.722 |
|  |  |  |  |  |  | definition 3: RECIST-defined PD and ΔTGR > 50 | 4/126 (3.2) |  |  | Median OS: HR, 2.82; 95% CI, 0.97– 8.17; *P*=.046 |
|  |  |  |  |  |  | definition 4: RECIST-defined PD, ≥ 10 mm increase in measurable lesions, tumor burden increase > 40%, and/or tumor burden increase > 20% with new lesions in ≥ 2 different organs | 14/126 (11.1) |  |  | Median OS: HR, 0.96; 95% CI, 0.407– 1.94; *P*=.967 |
|  |  |  |  |  | new lesions included in SLD | definition 5: RECIST-defined PD and TGR ratio ≥ 2 (on-treatment vs. pre-treatment) | 13/126 (10.3) |  |  | Median OS: HR, 3.57; 95% CI, 1.63– 7.82; *P*=.001 |
|  |  |  |  |  |  | definition 6: RECIST-defined PD and TGK ratio ≥ 2 (on-treatment vs. pre-treatment) | 14/126 (11.1) | *SMARCA2* mutation (*P*=.041), *MSH6* mutation (negative association; *P*=.039), alterations in the *APC* signaling pathway (*AMER1*, *APC*, *AXIN1*, *CDH1*, *CTNNB1*, *HNF1A*, *NF2*, *RNF43*, and *SOX9*; *P*=.021) |  | Median OS: HR, 2.30; 95% CI, 1.11– 4.78; *P*=.021 |
|  |  |  |  |  |  | definition 7: RECIST-defined PD and ΔTGR > 50% | 8/126 (6.3) | NA |  | Median OS: HR, 3.71; 95% CI, 1.54– 8.93; *P*=.002 |
| Maesaka et al,^11^ 2022 (Japan) | multicenter prospective study | October 2020 to May 2021 | HCC | PD-L1 inhibitor and VEGF inhibitor combination therapy | definition 1: RECIST-defined PD and TGR ratio and TGK ratio ≥ 2 (on-treatment vs. pre-treatment) | | 9/88 (12.5) | univariate: larger intrahepatic tumor size (median, 45 vs 20mm; *P*=.007), number of intrahepatic lesions (>5; *P*=.012), higher AFP (median, 793 vs 9 ng/mL; *P*=.005); higher LDH (median, 280 vs 194 U/L; *P*=.022)  multivariate: higher NLR (≥3; *P*=.019) | Median OS (definition 1): 4.3 mo vs. not reached (*P*<.001) | NA |
|  |  |  |  |  | definition 2^d^: RECIST-defined PD and TGR ratio ≥ 2 (on-treatment vs. pre-treatment) | | 9/88 (10.2) | NA | NA |  |
|  |  |  |  |  | definition 3: RECIST-defined PD and TGK ratio ≥ 2 (on-treatment vs. pre-treatment) | | 11/88 (10.2) |  |  |  |
| Takahashi et al,^35^ 2022 (Japan) | multicenter prospective study | March 2018 to August 2019 | AGC | PD-1 inhibitor monotherapy | RECIST-defined PD and TGR ratio ≥ 2 (on-treatment vs. pre-treatment) | | 45/219 (20.5) | NA | median period from first evaluation to death: HR 1.05 (95% CI 0.72–1.53, *P*=.8) | Median period from first evaluation to death: HR 1.77 (95% CI 1.25–2.51, *P*=.001) |
| Klemen et al,^36^ 2022 (USA) | single-center retrospective study (clinical trials data) | 2015 to 2019 | sarco-ma | PD-1 inhibitor monotherapy or in combination (with CTLA-4 inhibitor, oncolytic virus therapy, IL-2 pathway inhibitor, or IDO1 inhibitor) | RECIST-defined PD and ΔTGR > 50 | | 15/129 (11.6) | upregulated pathways compared to patients with progressive disease: glycolysis (*P*=.008), hypoxia (*P*=.033), and oxidative phosphorylation (*P*<.001), IFNα and IFNγ response pathways (both *P*<.001) | NA | Median OS: 7.7 vs. 5.9 mo (*P=*.34)  Median PFS: 1.6 vs. 1.6 mo (*P*>.99) |
| Kang et al,^37^ 2022 (Korea) | single-center retrospective study | January 2018 to August 2020 | NSCLC | PD-1/PD-L1 inhibitor monotherapy | RECIST-based TGK ratio ≥ 2 | | 6/74 (8.1) | higher frequency of CD4⁺CD25⁺CD127loFoxP3⁺ Treg cells 7 days after treatment initiation (*P*=.024) | NA | NA |

Abbreviations: AFP, α-fetoprotein; AGC, advanced gastric cancer; ANC, absolute neutrophil count; CD, cluster of differentiation; CRP, C-reactive protein; CTLA, cytotoxic T-lymphocyte-associated antigen 4; ECOG, Eastern Cooperative Oncology Group; HCC, hepatocellular carcinoma; HNSCC, head and neck squamous cell carcinoma; HPD, hyperprogressive disease; HR, hazard ratio; IDO1, indoleamine 2,3-dioxygenase 1; IL, interleukin; iRECIST, immune RECIST; irRC, immune-related response criteria; LDH, lactate dehydrogenase; NA, not available; NSCLC, non-small cell lung cancer; NLR, neutrophil-lymphocyte ratio; OS, overall survival; PD, progressive disease; PD-1, programmed cell death protein 1 ; PD-L1, programmed cell death 1 ligand 1; PFS, progression-free survival; PVTT, portal vein tumor thrombus; RCC, renal cell carcinoma; RECIST, response evaluation criteria in solid tumors; SLD, sum of longest diameters; TGK, tumor growth kinetics; TGR, tumor growth rate; TTF, time to treatment failure; UCC, urothelial cell carcinoma; VEGF, vascular endothelial growth factor

^a^Conference abstracts; all other studies were full-text articles.

^b^Studies that provided re-analyzed or adjusted incidences.

^c^Studies whose incidences were readjusted based on additional information provided in the text.

^d^The definition and the corresponding incidence that were used to represent the study in the meta-analysis.

**Table S4.** Studies excluded from full-text review, with reasons

| Study | Title | Reason for exclusion |
| --- | --- | --- |
| Cowzer 2020^38^ | MDM2 amplification and hyperprogression following treatment with immune checkpoint inhibitors in advanced non-small cell lung cancer | difference in study design |
| Gonzalez Espinoza 2020^39^ | Predictive biomarkers for hyper progression in response to immune checkpoint inhibitors therapy: Analysis of somatic alterations by NGS | difference in study design |
| Singavi 2017^40^ | Predictive biomarkers for hyper-progression (HP) in response to immune checkpoint inhibitors (ICI)-analysis of somatic alterations (SAs) | difference in study design |
| Singla 2021^41^ | Hyperprogression after Immunotherapy: Nivolumab. Analysis of Imaging Findings Associated with Hyperprogression and Tumor Growth Kinetics | difference in methodology of HPD evaluation |
| Nakamoto 2021^42^ | Imaging Characteristics and Diagnostic Performance of 2-deoxy-2-[(18)F]fluoro-D-Glucose PET/CT for Melanoma Patients Who Demonstrate Hyperprogressive Disease When Treated with Immunotherapy | difference in methodology of HPD evaluation |
| Kato 2017^43^ | Hyperprogressors after Immunotherapy: Analysis of Genomic Alterations Associated with Accelerated Growth Rate | difference in methodology of HPD evaluation |
| Li 2021^44^ | The genomic characteristics of different progression patterns in advanced non-small cell lung cancer patients treated with immune checkpoint inhibitors | difference in methodology of HPD evaluation |
| Choi 2020^45^ | Factors predicting hyperprogression in patients with advanced ovarian cancer receiving anti-programmed cell death 1-therapy | difference in methodology of HPD evaluation |
| Park 2020^46^ | Hyperprogressive disease and its clinical impact in patients with recurrent and/or metastatic head and neck squamous cell carcinoma treated with immune-checkpoint inhibitors: Korean cancer study group HN 18-12 | difference in methodology of HPD evaluation |
| Honjo 2018^47^ | Experience on hyperprogression disease (HPD) by immunotherapy for lung cancer | difference in methodology of HPD evaluation |
| Deng 2022^48^ | Real-world outcomes of patients with advanced intrahepatic cholangiocarcinoma treated with programmed cell death protein-1-targeted immunotherapy | difference in methodology of HPD evaluation |
| Okamoto 2020^49^ | Overall survival and PD-L1 expression in patients with recurrent or metastatic head and neck cancer treated with nivolumab | difference in methodology of HPD evaluation |
| Kanazu 2018^50^ | Hyperprogressive disease in patients with non-small cell lung cancer treated with nivolumab: A case series | insufficient data |
| Ghiglione 2019^51^ | Patterns and outcomes related to rapid progressive disease in a cohort of advanced solid tumours treated with immune checkpoint inhibitors (ICIs) | insufficient data |
| Yang 2021^52^ | Prevalence of hyperprogressive disease (HPD) mutations and correlations to immune-related biomarkers in a large pan-cancer Chinese cohort | insufficient data |
| Chen 2020^53^ | ctDNA Concentration, MIKI67 Mutations and Hyper-Progressive Disease Related Gene Mutations Are Prognostic Markers for Camrelizumab and Apatinib Combined Multiline Treatment in Advanced NSCLC | insufficient data |
| Boutin 2019^54^ | REAL WORLD EFFICACY AND TOXICITY OF ANTI-PD1 TREATMENT IN ELDERLY PATIENTS WITH ADVANCED NON-SMALL CELL LUNG CANCER (NSCLC) | insufficient data |
| Español-Rego 2021^55^ | A phase I-II multicenter trial with avelumab plus autologous dendritic cell vaccine in pre-treated mismatch repair-proficient (MSS) metastatic colorectal cancer patients. GEMCAD 16-02 (AVEVAC trial) | insufficient data |
| Yu 2020^56^ | Anti-PD-1 antibody monotherapy or anti-PD-1 antibody combination with chemotherapy treated nonsmall cell lung cancer (NSCLC) patients with EGFR mutation: A retrospective analysis | insufficient data |
| Raggi 2018^57^ | Apache: An open label, randomized, phase 2 study of durvalumab (Durva), alone or in combination with tremelimumab (Treme), in patients (pts) with advanced germ cell tumors (GCT): Results at the end of first stage | insufficient data |
| Alcaraz Sanchez 2021^58^ | Atezolizumab in non-small cell lung cancer: Effectiveness and safety real world data study | insufficient data |
| Nosaki 2018^59^ | Clinical Background and Response to Chemotherapy in NSCLC Patients with MET Exon14 Skipping Mutation or High MET Gene Copy Number | insufficient data |
| Lau 2020^60^ | Clinical outcome and toxicity for immunotherapy treatment in metastatic cancer patients | insufficient data |
| Rapposelli 2021^61^ | Heterogeneity of Response and Immune System Activity during Treatment with Nivolumab in Hepatocellular Carcinoma: Results from a Single-Institution Retrospective Analysis | insufficient data |
| Boileve 2017^62^ | Immune checkpoint inhibitors following targeted therapies in MITF family translocation renal cell carcinomas | insufficient data |
| Chen 2020^63^ | Novel immunotherapy combinations for betel-nuts related HNSCC: One institutional experience in Taiwan | insufficient data |
| Rubio 2019^64^ | P1.04-16 Early Antibiotic Use Affects the Efficacy of First Line Immunotherapy in Lung Cancer Patients but Route of Administration Seems to be Decisive | insufficient data |
| Gunduz 2019^65^ | P2.01-57 New Prognostic Markers in Patients with Lung Cancer Treated with Immunoterapy: NLR and PLR | insufficient data |
| Rubio 2019^66^ | P2.01-98 Neutrophil-Platelet Score (NPS), a Predictive Systemic Inflammation Score for Pembrolizumab in First Line of Advanced NSCLC Patients | insufficient data |
| Middleton 2018^67^ | Pembrolizumab in performance status 2 patients with non-small cell lung cancer (NSCLC): Results of the PePS2 trial | insufficient data |
| Wrangle 2021^68^ | Preliminary data from QUILT 3.055: A phase 2 multi-cohort study of N803 (IL-15 superagonist) in combination with checkpoint inhibitors (CPI) | insufficient data |
| Taugner 2021^69^ | Real-world prospective analysis of treatment patterns in durvalumab maintenance after chemoradiotherapy in unresectable, locally advanced NSCLC patients | insufficient data |
| Bruixola 2018^70^ | Safety and efficacy of nivolumab (nivo) in platinum-refractory recurrent/metastastic head and neck squamous cell (PR R/M HNSCC) patients (pts): Real-life experience | insufficient data |
| Granados 2018^71^ | Survival with nivolumab therapy in recurrent/advanced squamous cell head and neck carcinoma. A single center experience | insufficient data |
| Plimack 2021^72^ | A Phase II trial of guadecitabine (G) plus atezolizumab (A) in patients with metastatic urothelial carcinoma (UC) progressing after initial checkpoint inhibitor therapy | insufficient data |
| Mollica 2021^73^ | Tumor growth rate decline despite progressive disease may predict improved nivolumab treatment outcome in mrcc: When recist is not enough | insufficient data |
| Tunali 2019^74^ | Novel clinical and radiomic predictors of rapid disease progression phenotypes among lung cancer patients treated with immunotherapy: An early report | modified tumor dynamics-based definition |
| Trotier 2021^75^ | Genetic and molecular analysis of solid tumors with hyperprogressive disease after treatment with immunotherapy | modified tumor dynamics-based definition |
| Gomez 2019^76^ | P2.04-77 Hyperprogression with Immunotherapy in Metastatic Non-Small Cell Lung Cancer: Hôpital Charles-LeMoyne Experience | modified tumor dynamics-based definition |
| Kim 2021^77^ | The implications of clinical risk factors, CAR index, and compositional changes of immune cells on hyperprogressive disease in non-small cell lung cancer patients receiving immunotherapy | modified tumor dynamics-based definition |
| Lee 2021^78^ | Characterization of hyperprogressive disease in patients with advanced cancer treated with anti-PD-1 inhibitor: A multicenter retrospective study | modified tumor dynamics-based definition |
| Vaidya 2020^79^ | Novel, non-invasive imaging approach to identify patients with advanced non-small cell lung cancer at risk of hyperprogressive disease with immune checkpoint blockade | modified tumor dynamics-based definition |
| He 2021^80^ | CT-Based Peritumoral and Intratumoral Radiomics as Pretreatment Predictors of Atypical Responses to Immune Checkpoint Inhibitor Across Tumor Types: A Preliminary Multicenter Study | modified tumor dynamics-based definition |
| Takahashi 2019^81^ | Neutrophil-to-Lymphocyte ratio as a predictive factor for hyperprogressive disease in NSCLC patients treated with immune checkpoint inhibitor | modified tumor dynamics-based definition |
| Miyama 2021^82^ | Squamous differentiation is a potential biomarker predicting tumor progression in patients treated with pembrolizumab for urothelial carcinoma | modified tumor dynamics-based definition |
| Han 2019^83^ | Dynamic clonality of T cell receptor differentiate atypical progression in NSCLC patients treated with PD-1/PD-L1inhibitors | modified tumor dynamics-based definition |
| Zalcman 2019^84^ | Second/third-line nivolumab vs nivo plus ipilimumab in malignant pleural mesothelioma: Long-term results of IFCT-1501 MAPS2 phase IIR trial with a focus on hyperprogression (HPD) | modified tumor dynamics-based definition |
| Alfieri 2019^85^ | Evaluating hyperprogressive disease (HPD) in head and neck squamous cell carcinoma (HNSCC) patients treated with immune checkpoint inhibitors (ICI) | modified tumor dynamics-based definition |
| Paydary 2020^86^ | Hyperprogression on immune checkpoint inhibitors: A single institution, real-world retrospective analysis | modified tumor dynamics-based definition |
| Ferrara 2021^87^ | First-line platinum-based chemotherapy combined with PD-1/PD-l1 inhibitors (ICI) prevents hyperprogression in non-small cell lung cancer (NSCLC) patients by reducing circulating immature neutrophils | modified tumor dynamics-based definition |
| Xiao 2021^88^ | Lung metastasis and lymph node metastasis are risk factors for hyperprogressive disease in primary liver cancer patients treated with immune checkpoint inhibitors | not a tumor dynamics-based definition |
| Forschner 2020^89^ | MDM2, MDM4 and EGFR Amplifications and Hyperprogression in Metastatic Acral and Mucosal Melanoma | not a tumor dynamics-based definition |
| Abbas 2019^90^ | Hyperprogression after immunotherapy | not a tumor dynamics-based definition |
| Costantini 2018^91^ | Hyper-progressive disease in patients with advanced non-small cell lung cancer (NSCLC) treated with nivolumab (nivo) | not a tumor dynamics-based definition |
| Castello 2020^92^ | Hyperprogressive Disease in Patients with Non-Small Cell Lung Cancer Treated with Checkpoint Inhibitors: The Role of (18)F-FDG PET/CT | not a tumor dynamics-based definition |
| Kang 2022^93^ | Assessment of hyperprogression versus the natural course of disease development with nivolumab with or without ipilimumab versus placebo in phase III, randomized, controlled trials | not a tumor dynamics-based definition |
| Choi 2020^94^ | Prediction model for hyperprogressive disease in non-small cell lung cancer treated with immune checkpoint inhibitors | not a tumor dynamics-based definition |
| Perna 2018^95^ | Clinico-radiological pattern of response to nivolumab in stage IV NSCL: A real life experience over two years | not a tumor dynamics-based definition |
| ale Tadesse 2020^96^ | Molecular alterations with hyperprogression in lung cancer patients treated with immune checkpoint inhibitors in a large health system | not a tumor dynamics-based definition |
| ale Tadesse 2021^97^ | Genomic markers associated with hyperprogression in patients with lung cancer treated with immune checkpoint inhibitors | not a tumor dynamics-based definition |
| Rimola 2021^98^ | Radiological response to nivolumab in patients with hepatocellular carcinoma: A multicenter analysis of real-life practice | not a tumor dynamics-based definition |
| Reck 2019^99^ | Analysis of tumour hyperprogression (HP) with nivolumab (Nivo) in randomized, placebo (Pbo)-controlled trials | not a tumor dynamics-based definition |
| Colle 2020^100^ | Hyperprogression in 'real world' advanced melanoma patients treated by anti-PD1 | not a tumor dynamics-based definition |
| Decatris 2020^101^ | Exploratory analysis of factors associated with hyperprogression in advanced non-small cell lung cancer (NSCLC) treated with PD1/PDL1 inhibitors | not a tumor dynamics-based definition |
| Dey 2020^102^ | Hyperprogression in cancer patients on immunotherapeutic agents | not a tumor dynamics-based definition |
| Ruiz-Patiño 2020^103^ | Immunotherapy at any line of treatment improves survival in patients with advanced metastatic non-small cell lung cancer (NSCLC) compared with chemotherapy (Quijote-CLICaP) | not a tumor dynamics-based definition |
| Yilmaz 2021^104^ | Atypical response patterns in metastatic melanoma and renal cell carcinoma patients treated with nivolumab: A single center experience | not a tumor dynamics-based definition |
| Jin 2021^105^ | Anti-PD1 checkpoint inhibitor with or without chemotherapy for patients with recurrent and metastatic nasopharyngeal carcinoma | not a tumor dynamics-based definition |
| Matos 2018^106^ | Incidence and clinical implications of a new definition of hyperprogression (HPD) with immune checkpoint inhibitors (ICIs) in patients treated in phase 1 (Ph1) trials | not a tumor dynamics-based definition |
| Lee 2019^107^ | Hyperprogressive disease after two cycles of immunotherapy in HER-2 positive metastatic gastric cancer | not a tumor dynamics-based definition |
| Giusti 2019^108^ | CDKN2A/B gene loss and MDM2 alteration as a potential molecular signature for hyperprogressive disease in advanced NSCLC: A next-generation-sequencing approach | not a tumor dynamics-based definition |
| Fricke 2020^109^ | Alterations in STK11 to limit response to immune checkpoint inhibitors in lung cancer | not a tumor dynamics-based definition |
| Jang 2021^110^ | P75.13 Hyperprogressive Disease in Non-Small Cell Lung Cancer on pd-1 Inhibitor. | not a tumor dynamics-based definition |
| Ayers 2021^111^ | Analysis of Real-World Data to Investigate the Impact of Race and Ethnicity on Response to Programmed Cell Death-1 and Programmed Cell Death-Ligand 1 Inhibitors in Advanced Non-Small Cell Lung Cancers | not a tumor dynamics-based definition |
| Petrova 2020^112^ | Sarcopenia and high NLR are associated with the development of hyperprogressive disease after second-line pembrolizumab in patients with non-small-cell lung cancer | not a tumor dynamics-based definition |
| Feng 2018^113^ | Evaluating the occurrence of early tumor progression (ETP) in patients with gastric cancer treated with nivolumab versus placebo | not a tumor dynamics-based definition |
| Middleton 2020^114^ | Pembrolizumab in patients with non-small-cell lung cancer of performance status 2 (PePS2): a single arm, phase 2 trial | not a tumor dynamics-based definition |
| Kawabata 2020^115^ | Real-world efficacy and biomarker of nivolumab for advanced gastric cancer | overlapping cohort |
| Aoki 2019^116^ | The hyperprogressive disease during nivolumab treatment or irinotecan treatment in patients with advanced gastric cancer | overlapping cohort |
| Zuazo 2021^117^ | 1354P Comparison of different hyperprogressive disease criteria in non-small cell lung cancer patients treated with immunotherapy and correlation with somatic mutations in driver genes | overlapping cohort |
| Arasanz 2019^118^ | A burst of highly differentiated CD4 TL identifies a subset of fast progressors, and correlates with hyperprogressive disease in NSCLC patients treated with ICI | overlapping cohort |
| Ayala De Miguel 2019^119^ | Hyperprogressive disease in patients with advanced non-small cell lung cancer treated with immune checkpoint inhibitors | overlapping cohort |
| Economopoulou 2019^120^ | Clinical implications of hyperprogression with immune checkpoint inhibitors in patients with head and neck squamous cell carcinoma (HNSCC) | overlapping cohort |
| Psyrri 2019^121^ | Predictive biomarkers for response to nivolumab in head and neck squamous cell carcinoma (HNSCC) (NCT#03652142) | overlapping cohort |
| Lo Russo 2019^122^ | Antibody-Fc/FcR Interaction on Macrophages as a Mechanism for Hyperprogressive Disease in Non-small Cell Lung Cancer Subsequent to PD-1/PD-L1 Blockade | overlapping cohort |
| Kas 2020^1^ | Clarification of Definitions of Hyperprogressive Disease During Immunotherapy for Non-Small Cell Lung Cancer | overlapping cohort |
| Ferrara 2020^123^ | Comparison of fast-progression, hyperprogressive disease, and early deaths in advanced non-small-cell lung cancer treated with PD-1/PD-L1 inhibitors or chemotherapy | overlapping cohort |
| Abbar 2021^124^ | Definitions, outcomes, and management of hyperprogression in patients with non-small-cell lung cancer treated with immune checkpoint inhibitors | overlapping cohort |
| Ferrara 2019^125^ | Fast-progression (FP), hyper-progression (HPD) and early deaths (ED) in advanced non-small cell lung cancer (NSCLC) patients (pts) upon PD-(L)-1 blockade (IO) | overlapping cohort |
| Ferrara 2017^126^ | Hyperprogressive disease (HPD) is frequent in non-small cell lung cancer (NSCLC) patients (pts) treated with anti PD1/PD-L1 monoclonal antibodies (IO) | overlapping cohort |
| Champiat 2017^3^ | Hyperprogressive Disease Is a New Pattern of Progression in Cancer Patients Treated by Anti-PD-1/PD-L1 | overlapping cohort |
| Ferrara 2017^127^ | MA 10.11 Hyperprogressive Disease (HPD) Is Frequent in Non-Small Cell Lung Cancer (NSCLC) Patients (Pts) Treated with Anti PD1/PD-L1 Agents (IO) | overlapping cohort |
| Giusti 2019^128^ | CDKN2A/B gene loss and MDM2 alteration as a potential molecular signature for hyperprogressive disease in advanced NSCLC: A nextgeneration-sequencing approach. | overlapping cohort |
| De Morais 2020^129^ | Comparative assessment of different radiological criteria to identify paradoxical hyperprogression (HPD) to IO drugs | overlapping cohort |
| Lu 2019^130^ | Immunotherapy combinations for betel-nuts related HNSCC: one institutional experiences in Taiwan | overlapping cohort |
| Chen 2018^131^ | Possible CDK4/6 inhibitor use in betel-nuts related head and neck squamous cell carcinoma(HNSCC) | overlapping cohort |
| Hwang 2019^132^ | Hyperprogressive disease (HPD) in genitourinary (GU) cancer patients treated with PD-1/PD-L1 inhibitors | overlapping cohort |
| Kanjanapan 2018^133^ | Hyperprogressive disease (HPD) in early phase immunotherapy (IO) trials | overlapping cohort |
| Tan 2019^134^ | Hyperprogressive disease in advanced triplenegative breast cancer (aTNBC) treated with immunotherapy (IO) | overlapping cohort |
| Al-ezzi 2019^135^ | Impact of immunotherapy and targeted therapy on tumour growth rate in sarcoma | overlapping cohort |
| Chon 2020^136^ | Hyperprogressive disease during PD-1 blockade in patients with advanced hepatocellular carcinoma | overlapping cohort |
| Kim 2019^137^ | OA14.07 Clinical and Genetic Characterization of Hyperprogression Based on Volumetry in Advanced NSCLC Treated with Immunotherapy | overlapping cohort |
| Kim 2021^138^ | P14.25 Immune Cell Profiling of Hyperprogressive Disease in Patients with Non-Small Cell Lung Cancer Treated with Anti-PD-1/PD-L1 Antibodies | overlapping cohort |
| Kim 2019^139^ | Comprehensive Clinical and Genetic Characterization of Hyperprogression Based on Volumetry in Advanced Non-Small Cell Lung Cancer Treated With Immune Checkpoint Inhibitor | overlapping cohort |
| Kim 2018^140^ | Hyperprogression after immunotherapy: Clinical implication and genomic alterations in advanced non-small cell lung cancer patients (NSCLC) | overlapping cohort |
| Ku 2021^141^ | Tumor infiltrated immune cell types support distinct immune checkpoint inhibitor outcomes in patients with advanced non-small cell lung cancer | overlapping cohort |
| Song 2021^142^ | Evaluation of Response to Immune Checkpoint Inhibitors Using a Radiomics, Lesion-Level Approach | overlapping cohort |
| Lee 2019^143^ | P2.01-46 The Efficacy and Safety of 2nd-Line Nivolumab for Non-Small Cell Lung Cancer in Real-World Practice with Emphasis on Hyperprogession | overlapping cohort |
| Kim 2019^144^ | A new pattern called hyperprogression when using immune checkpoint blockers in real world | overlapping cohort |
| Kim 2019^145^ | Clinical implication of inflammation-based serologic biomarkers and tissue biomarkers on hyperprogression in NSCLC patients receiving immune checkpoint blockers in real world | overlapping cohort |
| Kim 2019^146^ | Clinical implication of multiplex IHC and serologic biomarkers on hyperprogression in NSCLC patients receiving immune checkpoint blockers in real world | overlapping cohort |
| Kim 2018^147^ | Hyperprogression and Pseudoprogression in Patients with Non-Small Cell Lung Cancer on Checkpoint Blocking Immunotherapy | overlapping cohort |
| Kim 2020^148^ | Multiplex immunohistochemistry accurately defines the immune compositional change of tumor microenvironment to predict hyperprogressivedisease | overlapping cohort |
| Klemen 2021^149^ | 1527MO Biomarkers of response and hyperprogression in patients with sarcoma treated with checkpoint blockade | overlapping cohort |
| Zhong 2020^150^ | The genomic characterization of different progression patterns in advanced lung cancer (LC) patients upon immune checkpoint inhibitor (ICI) treatment | overlapping cohort |
| Ferrara 2020^151^ | Circulating and tumor-associated neutrophil subtypes discriminate hyperprogressive disease (HPD) from conventional progression (PD) upon immune checkpoint inhibitors (ICI) in advanced nonsmall cell lung cancer (NSCLC) patients (pts) and in vivo models | overlapping cohort |
| Farè 2018^152^ | Hyperprogression during immuno-checkpoint inhibitors (ICIs): A clinically significant problem? | overlapping cohort |
| Alfieri 2019^153^ | Hyperprogressive disease (HPD) in head and neck squamous cell carcinoma (HNSCC) patients treated with immune checkpoint inhibitors (ICI) | overlapping cohort |
| Ferrara 2020^154^ | Hyperprogressive disease (HPD) upon first-line PD-1/PD-L1 inhibitors (ICI) as single agent or in combination with platinum-based chemotherapy in non-small cell lung cancer (NSCLC) patients (pts) | overlapping cohort |
| Lo Russo 2019^155^ | Hyperprogressive disease in advanced non-small cell lung cancer patients treated with immunotherapy | overlapping cohort |
| Ferrara 2021^156^ | Immunometabolism of circulating neutrophils in hyperprogressive disease (HPD) upon first-line PD-1/PD-L1 inhibitors (ICI) alone or in combination with platinum-based chemotherapy (PCT) in non-small cell lung cancer (NSCLC) patients (pts) | overlapping cohort |
| de Braud 2018^157^ | Is “hyper-progression” a relevant clinical item for patient with solid tumours candidate to check-point inhibitor treatment? | overlapping cohort |
| Lo Russo 2019^158^ | OA14.06 Hyperprogressive Disease in Advanced Non–Small Cell Lung Cancer Patients Treated with Immune Checkpoint Inhibitors | overlapping cohort |
| Sozzi 2018^159^ | PD-1 Blockade Promotes Hyperprogressive Disease in NSCLC Through Macrophages Activation via Antibody-Fc/FcR Interaction | overlapping cohort |
| Suarez 2019^160^ | Hyperprogressive disease in patients with metastatic genitourinary tumors treated with immune checkpoint inhibitors | overlapping cohort |
| Freixinos 2018^161^ | Immune profile and outcomes of patients (pts) with gynecological malignancies (GYN) enrolled in early phases immunotherapy (IO) trials | overlapping cohort |
| Garcia 2018^162^ | Refining criteria of hyperprogression (HPD) with immune checkpoint inhibitors (ICIs) to improve clinical applicability | overlapping cohort |
| Ortega Franco 2017^163^ | Does hyper-progression exist among head and neck cancer patients treated with immunotherapy? | overlapping cohort |
| Nakamoto 2020^164^ | Imaging characteristics and diagnostic performance of (18)F-FDG PET/CT for melanoma patients who demonstrate hyperprogressive disease when treated with immunotherapy | overlapping cohort |
| Petrova 2020^165^ | High neutrophil to lymphocyte ratio as a predictor for hyperprogressive disease in patients with metastatic non-small cell lung cancer treated with pembrolizumab as a second line | overlapping cohort |
| Necchi 2018^166^ | APACHE: An open label, randomized, phase II study of Durvalumab (Durva), alone or in combination with Tremelimumab (Treme), in patients (pts) with advanced germ cell tumors (GCT): Results at the end of first stage | overlapping cohort |
| Refae 2018^167^ | Host immunogenetics and hyperprogression under PD1/PD-L1 checkpoint inhibitors | overlapping cohort |
| Simões Da Rocha^168^ 2018 | Radiological identification of rapid progressions in advanced NSCLC patients treated with nivolumab | overlapping cohort |
| Arrieta 2019^169^ | P1.04-81 Characterization of Hispanic Patients Who Experienced Hyperprogression During Treatment for Advanced NSCLC with Immunotherapy | overlapping cohort |
| Arrieta 2019^170^ | P2.23 Characterization of Hispanic Patients Who Experienced Hyperprogression During Treatment for Advanced NSCLC with Immunotherapy | overlapping cohort |
| Togashi 2018^171^ | Clinicopathological, genomic and immunological features of hyperprogressive disease during PD-1 blockade in gastric cancer patients | overlapping cohort |
| Sasaki 2018^172^ | Predictive factor of hyperprogressive disease during nivolumab in patients with advanced gastric cancer | overlapping cohort |
| Scheiner 2019^173^ | PD-1 targeted immunotherapy in advanced hepatocellular carcinoma: Efficacy and safety data from an international multicenter real-world cohort | overlapping cohort |
| Sugimoto 2019^174^ | Hyperprogressive disease (HPD) during nivolumab (Nivo) or irinotecan (IRI) as salvage line in patients with metastatic gastric cancer (MGC) | overlapping cohort |
| Sunakawa 2019^175^ | Interim analysis of an observational/translational study for nivolumab treatment in advanced gastric cancer: JACCRO GC-08 (DELIVER trial) | overlapping cohort |
| Ishiguro 2021^176^ | O1-4 Survival time of nivolumab treatment in advanced gastric cancer from real-world data of the DELIVER trial (JACCRO GC-08) | overlapping cohort |
| Kawabata 2020^115^ | Tumor response and growth rate of nivolumab treatment in advanced gastric cancer: Realworld data from a large observational/translational study, JACCRO GC-08 (deliver trial) | overlapping cohort |
| Sunakawa 2020^177^ | Updated analysis of DELIVER trial (JACCRO GC-08): A large observational/translational study of nivolumab treatment in advanced gastric cancer | overlapping cohort |
| Hagi 2020^178^ | Multicentre biomarker cohort study on the efficacy of nivolumab treatment for gastric cancer | overlapping cohort |
| Taugner 2021^179^ | Real-world analysis of treatment patterns and efficacy of durvalumab maintenance after chemoradiotherapy in NSCLC patients | overlapping cohort |
| Taugner 2021^180^ | Treatment patterns and efficacy of durvalumab maintenance after CRT in real-world NSCLC patients | overlapping cohort |
| Berge 2019^181^ | P1.09-21 Tumor Responses Based on Tumor Growth Rate During PD-1 Inhibitor Therapy in Advanced Non-Small-Cell Lung Cancer Patients | overlapping cohort |
| Tunali 2017^182^ | Epidemiologic and radiomic analysis of hyperprogressers of lung cancer patients treated with immunotherapy | overlapping cohort |
| Patil 2018^183^ | Pre-Therapy Radiomic Features Can Distinguish Hyperprogression from Other Response Patterns to PD1/PD-L1 Inhibitors in NSCLC | overlapping cohort |
| Ji 2020^184^ | Use of Radiomics to Predict Response to Immunotherapy of Malignant Tumors of the Digestive System | overlapping cohort |
| Lu 2019^185^ | Serological Markers Associated With Response to Immune Checkpoint Blockade in Metastatic Gastrointestinal Tract Cancer | overlapping cohort |
| Kamada 2019^186^ | PD-1(+) regulatory T cells amplified by PD-1 blockade promote hyperprogression of cancer | overlapping cohort |
| Ji 2019^187^ | Hyperprogression after immunotherapy in patients with malignant tumors of digestive system | overlapping cohort |
| Suzuki 2020^188^ | Hyperprogressive disease during nivolumab chemotherapy in metastatic gastric cancer: Multicenter retrospective study in Japan | overlapping cohort |
| Guiard 2022^189^ | Radiological patterns of tumour progression in patients treated with a combination of immune checkpoint blockers and antiangiogenic drugs | overlapping cohort |
| Saâda-Bouzid 2017^190^ | Hyperprogression during anti-PD-1/PD-L1 therapy in patients with recurrent and/or metastatic head and neck squamous cell carcinoma | overlapping cohort |
| Kadowaki 2022^191^ | Association of disease progression pattern during third-line chemotherapy with nivolumab with poor prognosis in advanced gastric cancer: A multicenter retrospective study in Japan | overlapping cohort |
| Kubota 2021^192^ | Rare Nivolumab-associated Super Hyper Progressive Disease in Patients With Advanced Gastric Cancer | overlapping cohort |
| Sugimoto 2018^193^ | Hyperprogression during nivolumab (Nivo) or irinotecan (IRI) as salvage-line in patients with metastatic gastric cancer | overlapping cohort |

**Table S5. Categorization of studies included in the meta-analysis according to the definition used**

|  | Criteria used to define HPD | Original source of this definition | Studies based on this definition^a^ | Differences from the original definition |
| --- | --- | --- | --- | --- |
| The definition includes a single metric describing the tumor growth kinetics | RECIST-defined PD and TGR ratio ≥ 2 (on-treatment vs. pre-treatment) | Champiat et al,^3^ 2017 | Aoki et al,^23^ 2019; Kanjanapan et al,^24^ 2019; Tang et al,^26^ 2019; ten Berge et al,^27^ 2019; Park et al,^19^ 2019^b^; Petrioli et al,^28^ 2020; Matos et al,^5^ 2020^b^; Kim et al^21^, 2020; Rocha et al,^32^ 2021^b^; Matsuo et al,^16^ 2021; Takahashi et al,^35^ 2022 | none |
|  |  |  | Zheng et al,^33^ 2021 | used iRECIST and RECIST criteria |
|  |  |  | Arasanz et al,^20^ 2020^c^ | used RECIST and irRC |
|  | RECIST-defined PD and ΔTGR > 50 | Ferrara et al,^4^ 2018 | Mazza et al,^22^ 2017; Scheiner et al,^25^ 2019; Ayala de Miguel,^8^ 2020; Chen et al,^7^ 2021; Klemen et al,^36^ 2022 | none |
|  | RECIST-based TGK ratio ≥ 2 (on-treatment vs. pre-treatment) | Saâda-Bouzid et al,^2^ 2017^c^ | Refae et al,^14^ 2020; Karabajakian et al,^12^ 2020; Gomes da Morais et al,^29^ 2020^b^; Economopoulou et al,^15^ 2021; Kang et al,^37^ 2022 | One of the definitions studied by Gomes da Morais et al,^29^ 2020 used a different threshold (ΔTGR > 100)^1^ |
|  |  |  | Wang et al,^17^ 2021^b^ | incorporated new lesions into the tumor burden |
|  | TTF < 2 mo, irRC-defined tumor burden increase > 50%, and progression pace increase > 2-fold | Kato et al,^43^ 2017 | Hwang et al,^6^ 2020 | used RECIST criteria; TGR ratio for "progression pace"; the development of extensive (> 10) new lesions also defined HPD |
|  |  |  | Schuiveling et al,^34^ 2021^b^ | used iRECIST criteria; TGR ratio for "progression pace” |
|  |  |  | Sasaki et al,^9^ 2019 | used RECIST criteria; excluded criterion of TTF < 2 mo; TGK ratio for "progression pace" |
| The definition includes more than one metric describing the tumor growth kinetics | RECIST-defined PD, TGR ratio and TGK ratio ≥ 2 (on-treatment vs. pre-treatment) | Combinations of definitions by Champiat et al^3^ and Saâda-Bouzid et al^2^ | Kim et al,^18^ 2019^b^; Kim et al,^30^ 2020^b^; Maesaka et al,^11^ 2022^b^ | none |
|  |  |  | Choi et al,^13^ 2021 | different threshold (TGR ratio and TGK ratio > 4) |
|  | RECIST-defined PD, TGR and TGK ratio ≥ 2 and ΔTGR > 50 (on-treatment vs. pre-treatment) | Combinations of definitions by Champiat et al,^3^ Saâda-Bouzid et al,^2^ and Ferrara et al^4^ | Kim et al,^10^ 2021^b^ | different threshold (TGK ratio and TGR ratio > 4, ΔTGR > 40) |
|  | TTF < 2 months, RECIST-defined PD, TGR ratio ≥ 2, and ΔTGR > 50 (on-treatment vs. pre-treatment) | Combinations of definitions by Kato et al,^43^ Champiat et al,^3^ and Ferrara et al^4^ | Zhang et al,^31^ 2021 | none |

Abbreviations: iRECIST, immune RECIST; irRC, immune-related response criteria; RECIST, response evaluation criteria in solid tumors; PD, progressive disease; TGK, tumor growth kinetics; TGR, tumor growth rate; TTF, time to treatment failure

^a^This column only includes studies which were included in the meta-analysis.

^b^Studies which explored more than one definition. For these studies, the definition which was preferred by the authors is represented in the table. If there were no preferences, the study has been placed in the category of the definition by Champiat et al.^3^

^c^RECIST was used to define target lesions for HPD evaluation. Saâda-Bouzid et al^2^ used irRECIST for a comparison of prognosis with RECIST. Arasanz et al^20^ applied irRC’s principle of confirming progression with a repeated and consecutive assessment in order to distinguish pseudoprogression from true PD.

**Table S6. Categorization of studies included in the meta-analysis according to the tumor type**

| Tumor type | Study |
| --- | --- |
| Advanced gastric cancer | Aoki et al,^23^ 2019; Sasaki et al,^9^ 2019; Takahashi et al,^35^ 2022 |
| Hepatocellular carcinoma | Choi et al,^13^ 2021; Kim et al,^10^ 2021; Maesaka et al,^11^ 2022; Scheiner et al,^25^ 2019; Zhang et al,^31^ 2021 |
| Mixed or other | Chen et al,^7^ 2021; Economopoulou et al,^15^ 2021; Gomes da Morais et al,^29^ 2020; Kanjanapan et al,^24^ 2019; Karabajakian et al,^12^ 2020; Klemen et al,^36^ 2022; Matos et al,^5^ 2020; Petrioli et al,^28^ 2020; Refae et al,^14^ 2020; Schuiveling et al,^34^ 2021; Tang et al,^26^ 2019; Wang et al,^17^ 2021 |
| Non-small cell lung cancer | Ayala de Miguel,^8^ 2020; Arasanz et al,^20^ 2020; Ferrara et al,^4^ 2018; Kang et al,^37^ 2022; Kim et al,^18^ 2019; Kim et al^21^, 2020; Kim et al,^30^ 2020; Matsuo et al,^16^ 2021; Park et al,^19^ 2019; Rocha et al,^32^ 2021; ten Berge et al,^27^ 2019 |
| Renal cell carcinoma | Hwang et al,^6^ 2020; Mazza et al,^22^ 2017; Zheng et al,^33^ 2021 |

**Table S7.** Quality of studies included in the meta-analysis according to the Newcastle-Ottawa Scale

| **Study** | **Selection of cohorts** | | | | **Comparability of cohorts** | **Outcome** | | | **Score** |
| --- | --- | --- | --- | --- | --- | --- | --- | --- | --- |
|  | Representat-iveness of the exposed  cohort | Selection of the non-exposed cohort | Ascertainment of exposure | Demonstration that outcome of interest was not present at start of study | Comparability of cohorts on the basis of the design  or analysis | Assessment of outcome | Adequacy of follow-up length | Adequacy of follow-up rate |  |
| Mazza et al 2017^22^ | ☆ | ☆ | ☆ | ☆ |  | ☆ | ☆ |  | 6 |
| Ferrara et al 2018^4^ | ☆ | ☆ | ☆ |  | ☆☆ | ☆ | ☆ |  | 7 |
| Aoki et al 2019^23^ | ☆ | ☆ | ☆ | ☆ | ☆☆ | ☆ | ☆ |  | 8 |
| Kanjanapan et al 2019^24^ | ☆ | ☆ | ☆ | ☆ | ☆☆ | ☆ | ☆ |  | 8 |
| Scheiner et al 2019^25^ | ☆ | ☆ | ☆ |  |  | ☆ | ☆ |  | 5 |
| Tang et al 2019^26^ | ☆ | ☆ | ☆ | ☆ |  | ☆ | ☆ |  | 6 |
| Sasaki et al 2019^9*^ | ☆ | ☆ | ☆ | ☆ | ☆☆ | ☆ | ☆ |  | 8 |
| Kim et al 2019^18^ | ☆ | ☆ | ☆ | ☆ | ☆☆ | ☆ | ☆ |  | 8 |
| ten Berge et al 2019^27^ | ☆ | ☆ | ☆ | ☆ |  | ☆ | ☆ |  | 6 |
| Park et al 2019^19^ | ☆ | ☆ | ☆ | ☆ | ☆☆ | ☆ | ☆ |  | 8 |
| Petrioli et al 2020^28^ | ☆ | ☆ | ☆ | ☆ | ☆☆ | ☆ | ☆ |  | 8 |
| Arasanz et al 2020^20^ | ☆ | ☆ | ☆ | ☆ | ☆☆ | ☆ | ☆ |  | 8 |
| Refae et al 2020^14^ | ☆ | ☆ | ☆ |  | ☆☆ | ☆ | ☆ |  | 7 |
| Hwang et al 2020^6^ | ☆ | ☆ | ☆ |  | ☆☆ | ☆ | ☆ |  | 7 |
| Matos et al 2020^5^ | ☆ | ☆ | ☆ |  | ☆☆ | ☆ | ☆ |  | 7 |
| Karabajakian et al 2020^12^ | ☆ | ☆ | ☆ |  | ☆☆ | ☆ | ☆ |  | 7 |
| Ayala de Miguel 2020^8^ | ☆ | ☆ | ☆ |  | ☆☆ | ☆ | ☆ |  | 7 |
| Kim et al 2020^21^ | ☆ | ☆ | ☆ | ☆ | ☆☆ | ☆ | ☆ |  | 8 |
| Gomes da Morais et al 2020^29^ | ☆ | ☆ | ☆ |  | ☆☆ | ☆ | ☆ |  | 7 |
| Kim et al 2020^30^ | ☆ | ☆ | ☆ | ☆ | ☆☆ | ☆ | ☆ |  | 8 |
| Zhang et al 2021^31^ | ☆ | ☆ | ☆ | ☆ | ☆☆ | ☆ | ☆ |  | 8 |
| Rocha et al 2021^32^ | ☆ | ☆ | ☆ | ☆ | ☆☆ | ☆ | ☆ |  | 8 |
| Economopoulou et al 2021^15^ | ☆ | ☆ | ☆ | ☆ | ☆☆ | ☆ | ☆ |  | 8 |
| Kim et al 2021^10^ | ☆ | ☆ | ☆ | ☆ | ☆☆ | ☆ | ☆ |  | 8 |
| Zheng et al 2021^33^ | ☆ | ☆ | ☆ | ☆ |  | ☆ | ☆ |  | 6 |
| Schuiveling et al 2021^34^ | ☆ | ☆ | ☆ |  | ☆☆ | ☆ | ☆ |  | 7 |
| Chen et al 2021^7^ | ☆ | ☆ | ☆ |  | ☆☆ | ☆ | ☆ |  | 7 |
| Matsuo et al 2021^16^ | ☆ | ☆ | ☆ |  |  | ☆ | ☆ |  | 5 |
| Choi et al 2021^13^ | ☆ | ☆ | ☆ |  | ☆☆ | ☆ | ☆ |  | 7 |
| Wang et al 2021^17^ | ☆ | ☆ | ☆ | ☆ | ☆☆ | ☆ | ☆ |  | 8 |
| Maesaka et al 2022^11^ | ☆ | ☆ | ☆ | ☆ | ☆☆ | ☆ | ☆ |  | 8 |
| Takahashi et al 2022^35^ | ☆ | ☆ | ☆ | ☆ | ☆☆ | ☆ | ☆ |  | 8 |
| Klemen et al 2022^36^ | ☆ | ☆ | ☆ | ☆ | ☆☆ | ☆ | ☆ |  | 8 |
| Kang et al 2022^37^ | ☆ | ☆ | ☆ |  | ☆☆ | ☆ | ☆ |  | 7 |

**Table S8.** Sensitivity analyses after excluding prospective cohort studies, low-quality studies (NOS<7), conference abstracts, or outliers.

|  | Incidence (%) | *Q* | *P*-value | *I^2^* (%) | 95% CI | 95% PI |
| --- | --- | --- | --- | --- | --- | --- |
| Main analysis | 12.4 | 119.32 | <.001 | 72.3 | 10.2 - 15.0 | 4.5 − 29.7 |
| Excluding prospective studies | 12.0 | 111.59 | <.001 | 73.1 | 9.7 - 14.8 | 4.2 - 30.0 |
| Excluding low-quality studies (NOS <7) | 13.7 | 105.05 | <.001 | 74.3 | 11.3 - 16.5 | 5.4 - 30.4 |
| Excluding conference abstracts | 13.2 | 106.34 | <.001 | 72.7 | 10.8 - 16.0 | 5.0 - 30.5 |
| Excluding outliers^a^ | 11.8 | 87.15 | <.001 | 64.4 | 9.8 - 14.0 | 5.0 - 25.1 |

^a^Outliers were identified as studies found in the upper right quadrant of the Baujat plot.

**Figure S1.** Contour-enhanced funnel plot for all studies included in the meta-analysis.


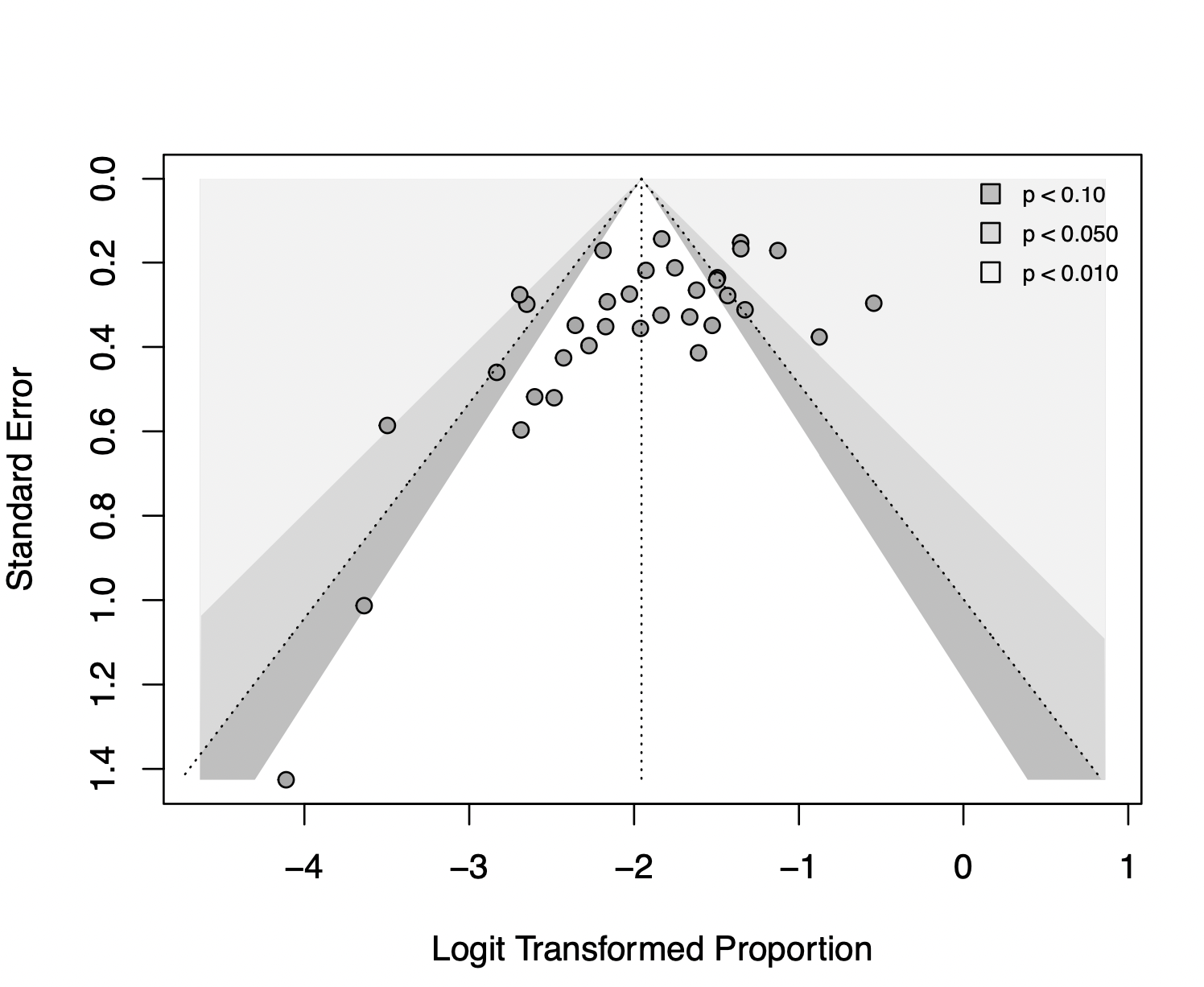


**Figure S2.** Baujat plot of studies included in the meta-analysis

**
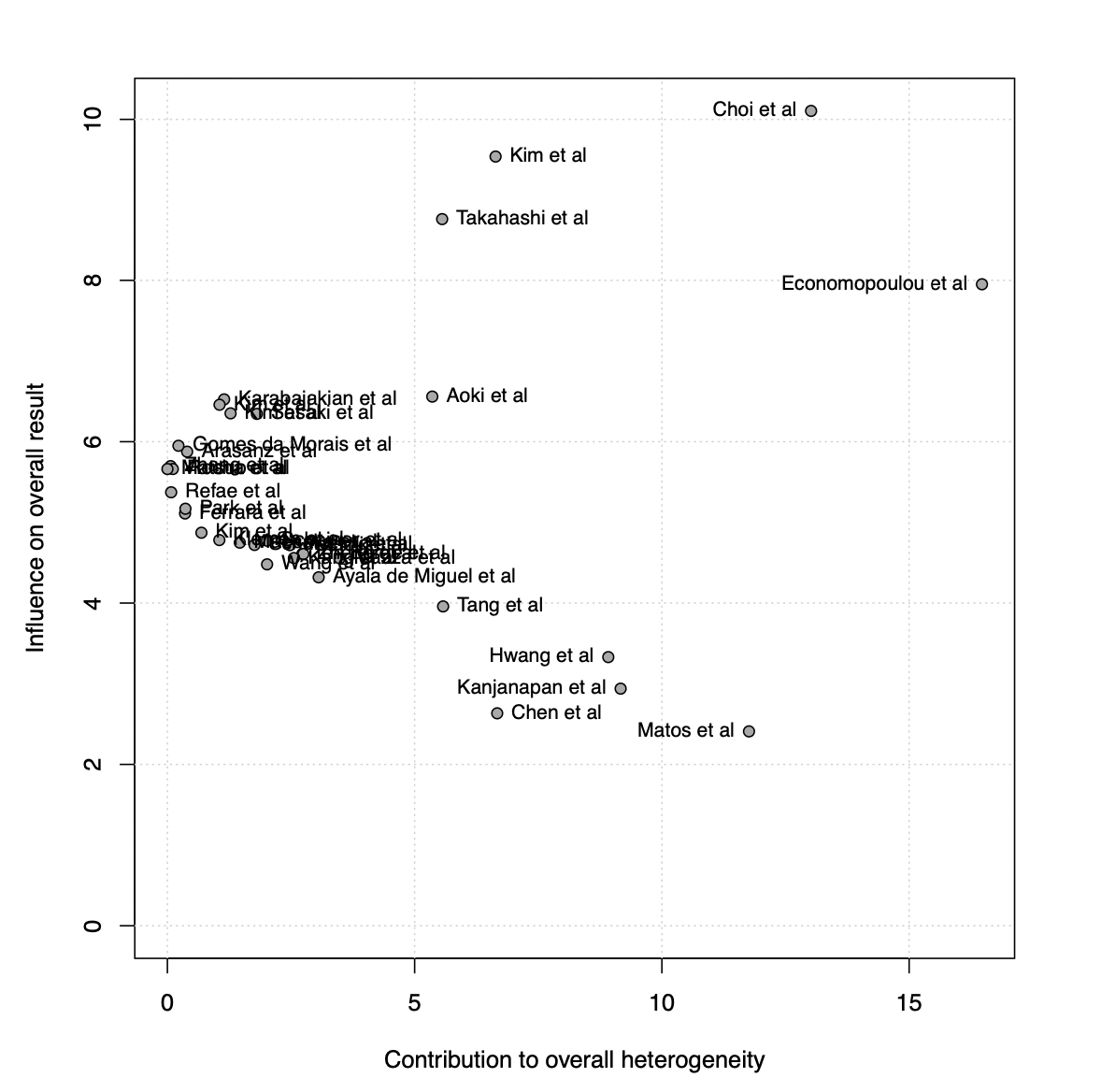
**

**Supporting Information References.**

1. Kas B, Talbot H, Ferrara R, et al. Clarification of Definitions of Hyperprogressive Disease During Immunotherapy for Non-Small Cell Lung Cancer. *JAMA Oncol* 2020; 6: 1039–1046.

2. Saâda-Bouzid E, Defaucheux C, Karabajakian A, et al. Hyperprogression during anti-PD-1/PD-L1 therapy in patients with recurrent and/or metastatic head and neck squamous cell carcinoma. *Ann Oncol Off J Eur Soc Med Oncol* 2017; 28: 1605–1611.

3. Champiat S, Dercle L, Ammari S, et al. Hyperprogressive Disease Is a New Pattern of Progression in Cancer Patients Treated by Anti-PD-1/PD-L1. *Clin Cancer Res Off J Am Assoc Cancer Res* 2017; 23: 1920–1928.

4. Ferrara R, Mezquita L, Texier M, et al. Hyperprogressive Disease in Patients With Advanced Non–Small Cell Lung Cancer Treated With PD-1/PD-L1 Inhibitors or With Single-Agent Chemotherapy. *JAMA Oncol* 2018; 4: 1543–1552.

5. Matos I, Martin-Liberal J, García-Ruiz A, et al. Capturing Hyperprogressive Disease with Immune-Checkpoint Inhibitors Using RECIST 1.1 Criteria. *Clin Cancer Res Off J Am Assoc Cancer Res* 2020; 26: 1846–1855.

6. Hwang I, Park I, Yoon S-K, et al. Hyperprogressive Disease in Patients With Urothelial Carcinoma or Renal Cell Carcinoma Treated With PD-1/PD-L1 Inhibitors. *Clin Genitourin Cancer* 2020; 18: e122–e133.

7. Chen S, Gou M, Yan H, et al. Hyperprogressive Disease Caused by PD-1 Inhibitors for the Treatment of Pan-Cancer. *Dis Markers* 2021; 2021: e6639366.

8. Ayala de Miguel P, López Gallego J, Gorospe García I, et al. Hyperprogressive disease during treatment with immune checkpoint inhibitors in patients with advanced non-small cell lung cancer (NSCLC). *J Clin Oncol* 2020; 38: e21664–e21664.

9. Sasaki A, Nakamura Y, Mishima S, et al. Predictive factors for hyperprogressive disease during nivolumab as anti-PD1 treatment in patients with advanced gastric cancer. *Gastric Cancer Off J Int Gastric Cancer Assoc Jpn Gastric Cancer Assoc* 2019; 22: 793–802.

10. Kim CG, Kim C, Yoon SE, et al. Hyperprogressive disease during PD-1 blockade in patients with advanced hepatocellular carcinoma. *J Hepatol* 2021; 74: 350–359.

11. Maesaka K, Sakamori R, Yamada R, et al. Hyperprogressive disease in patients with unresectable hepatocellular carcinoma receiving atezolizumab plus bevacizumab therapy. *Hepatol Res* 2022; 52: 298–307.

12. Karabajakian A, Garrivier T, Crozes C, et al. Hyperprogression and impact of tumor growth kinetics after PD1/PDL1 inhibition in head and neck squamous cell carcinoma. *Oncotarget* 2020; 11: 1618–1628.

13. Choi W-M, Kim JY, Choi J, et al. Kinetics of the neutrophil-lymphocyte ratio during PD-1 inhibition as a prognostic factor in advanced hepatocellular carcinoma. *Liver Int Off J Int Assoc Study Liver* 2021; 41: 2189–2199.

14. Refae S, Gal J, Brest P, et al. Hyperprogression under Immune Checkpoint Inhibitor: a potential role for germinal immunogenetics. *Sci Rep* 2020; 10: 3565.

15. Economopoulou P, Anastasiou M, Papaxoinis G, et al. Patterns of Response to Immune Checkpoint Inhibitors in Association with Genomic and Clinical Features in Patients with Head and Neck Squamous Cell Carcinoma (HNSCC). *Cancers* 2021; 13: 286.

16. Matsuo N, Azuma K, Kojima T, et al. Comparative incidence of immune-related adverse events and hyperprogressive disease in patients with non-small cell lung cancer receiving immune checkpoint inhibitors with and without chemotherapy. *Invest New Drugs* 2021; 39: 1150–1158.

17. Wang Z, Liu C, Bai Y, et al. Redefine Hyperprogressive Disease During Treatment With Immune-Checkpoint Inhibitors in Patients With Gastrointestinal Cancer. *Front Oncol*; 11, https://www.frontiersin.org/article/10.3389/fonc.2021.761110 (2021, accessed 2 March 2022).

18. Kim CG, Kim KH, Pyo K-H, et al. Hyperprogressive disease during PD-1/PD-L1 blockade in patients with non-small-cell lung cancer. *Ann Oncol Off J Eur Soc Med Oncol* 2019; 30: 1104–1113.

19. Park C, Kim M, Oh I, et al. P1.04-70 Application of Tumor Kinetics for Evaluation of Hyperprogression in Immune Checkpoint Inhibitor Treatment for Non-Small Cell Lung Cancer. *J Thorac Oncol* 2019; 14: S469.

20. Arasanz H, Zuazo M, Bocanegra A, et al. Early Detection of Hyperprogressive Disease in Non-Small Cell Lung Cancer by Monitoring of Systemic T Cell Dynamics. *Cancers* 2020; 12: E344.

21. Kim SH, Choi C-M, Lee DH, et al. Clinical outcomes of nivolumab in patients with advanced non-small cell lung cancer in real-world practice, with an emphasis on hyper-progressive disease. *J Cancer Res Clin Oncol* 2020; 146: 3025–3036.

22. Mazza C, Arfi-Rouche J, Koscielny S, et al. Effect of nivolumab on tumor growth rate (TGR) in metastatic renal cell carcinoma (mRCC). *J Clin Oncol* 2017; 35: 481–481.

23. Aoki M, Shoji H, Nagashima K, et al. Hyperprogressive disease during nivolumab or irinotecan treatment in patients with advanced gastric cancer. *ESMO Open* 2019; 4: e000488.

24. Kanjanapan Y, Day D, Wang L, et al. Hyperprogressive disease in early-phase immunotherapy trials: Clinical predictors and association with immune-related toxicities. *Cancer* 2019; 125: 1341–1349.

25. Scheiner B, Kirstein MM, Hucke F, et al. Programmed cell death protein-1 (PD-1)-targeted immunotherapy in advanced hepatocellular carcinoma: efficacy and safety data from an international multicentre real-world cohort. *Aliment Pharmacol Ther* 2019; 49: 1323–1333.

26. Tang B, Chi Z, Sheng X, et al. Tumor growth rate as an early indicator of the efficacy of anti-PD-1 immunotherapy in advanced melanoma. *J Clin Oncol* 2019; 37: e21050–e21050.

27. ten Berge DMHJ, Hurkmans DP, den Besten I, et al. Tumour growth rate as a tool for response evaluation during PD-1 treatment for non-small cell lung cancer: a retrospective analysis. *ERJ Open Res* 2019; 5: 00179–02019.

28. Petrioli R, Mazzei MA, Giorgi S, et al. Hyperprogressive disease in advanced cancer patients treated with nivolumab: a case series study. *Anticancer Drugs* 2020; 31: 190–195.

29. Gomes da Morais AL, de Miguel M, Cardenas JM, et al. Comparison of radiological criteria for hyperprogressive disease in response to immunotherapy. *Cancer Treat Rev* 2020; 91: 102116.

30. Kim KH, Hur JY, Koh J, et al. Immunological Characteristics of Hyperprogressive Disease in Patients with Non-small Cell Lung Cancer Treated with Anti-PD-1/PD-L1 Abs. *Immune Netw* 2020; 20: e48.

31. Zhang L, Wu L, Chen Q, et al. Predicting hyperprogressive disease in patients with advanced hepatocellular carcinoma treated with anti-programmed cell death 1 therapy. *EClinicalMedicine* 2021; 31: 100673.

32. Rocha P, Ramal D, Ripoll E, et al. Comparison of Different Methods for Defining Hyperprogressive Disease in NSCLC. *JTO Clin Res Rep* 2021; 2: 100115.

33. Zheng B, Shin JH, Li H, et al. Comparison of Radiological Tumor Response Based on iRECIST and RECIST 1.1 in Metastatic Clear-Cell Renal Cell Carcinoma Patients Treated with Programmed Cell Death-1 Inhibitor Therapy. *Korean J Radiol* 2021; 22: 366–375.

34. Schuiveling M, Tonk EHJ, Verheijden RJ, et al. Hyperprogressive disease rarely occurs during checkpoint inhibitor treatment for advanced melanoma. *Cancer Immunol Immunother CII* 2021; 70: 1491–1496.

35. Takahashi Y, Sunakawa Y, Inoue E, et al. Real-world effectiveness of nivolumab in advanced gastric cancer: the DELIVER trial (JACCRO GC-08). *Gastric Cancer* 2022; 25: 235–244.

36. Klemen ND, Hwang S, Bradic M, et al. Long-term Follow-up and Patterns of Response, Progression, and Hyperprogression in Patients after PD-1 Blockade in Advanced Sarcoma. *Clin Cancer Res* 2022; OF1–OF9.

37. Kang DH, Chung C, Sun P, et al. Circulating regulatory T cells predict efficacy and atypical responses in lung cancer patients treated with PD-1/PD-L1 inhibitors. *Cancer Immunol Immunother CII* 2022; 71: 579–588.

38. Cowzer D, Blazkova S, Henry S, et al. MDM2 amplification and hyperprogression following treatment with immune checkpoint inhibitors in advanced non-small cell lung cancer. *Ann Oncol* 2020; 31: S1112–S1113.

39. Gonzalez Espinoza IRR, Cortés Escobar N, Ibarra Fernández R, et al. Predictive biomarkers for hyper progression in response to immune checkpoint inhibitors therapy: Analysis of somatic alterations by NGS. *Ann Oncol* 2020; 31: S288.

40. Singavi AK, Menon S, Kilari D, et al. Predictive biomarkers for hyper-progression (HP) in response to immune checkpoint inhibitors (ICI) - analysis of somatic alterations (SAs). *Ann Oncol*; 28.

41. Singla R, Gupta A, Batra U, et al. Hyperprogression after Immunotherapy: Nivolumab. Analysis of Imaging Findings Associated with Hyperprogression and Tumor Growth Kinetics. *Indian J Radiol Imaging* 2021; 31: 345–349.

42. Nakamoto R, C Zaba L, Rosenberg J, et al. Imaging Characteristics and Diagnostic Performance of 2-deoxy-2-[18F]fluoro-D-Glucose PET/CT for Melanoma Patients Who Demonstrate Hyperprogressive Disease When Treated with Immunotherapy. *Mol Imaging Biol* 2021; 23: 139–147.

43. Kato S, Goodman A, Walavalkar V, et al. Hyperprogressors after Immunotherapy: Analysis of Genomic Alterations Associated with Accelerated Growth Rate. *Clin Cancer Res Off J Am Assoc Cancer Res* 2017; 23: 4242–4250.

44. Li J, Xiang C, Wang Y, et al. The genomic characteristics of different progression patterns in advanced non-small cell lung cancer patients treated with immune checkpoint inhibitors. *Ann Transl Med* 2021; 9: 779.

45. Choi JY, Park JY, Lee SW, et al. Factors predicting hyperprogression in patients with advanced ovarian cancer receiving anti-programmed cell death 1-therapy. *Gynecol Oncol* 2020; 159: 154.

46. Park JH, Chun SH, Lee Y-G, et al. Hyperprogressive disease and its clinical impact in patients with recurrent and/or metastatic head and neck squamous cell carcinoma treated with immune-checkpoint inhibitors: Korean cancer study group HN 18–12. *J Cancer Res Clin Oncol* 2020; 146: 3359–3369.

47. Honjo O, Fujita A, Saikai T, et al. Experience on hyperprogression disease (HPD) by immunotherapy for lung cancer. *Respirology* 2018; 23: 163.

48. Deng M, Li S, Wang Q, et al. Real-world outcomes of patients with advanced intrahepatic cholangiocarcinoma treated with programmed cell death protein-1-targeted immunotherapy. *Ann Med* 2022; 54: 803–811.

49. Okamoto I, Sato H, Tsukahara K. Overall survival and PD-L1 expression in patients with recurrent or metastatic head and neck cancer treated with nivolumab. *Auris Nasus Larynx* 2020; 47: 676–686.

50. Kanazu M, Edahiro R, Krebe H, et al. Hyperprogressive disease in patients with non-small cell lung cancer treated with nivolumab: A case series. *Thorac Cancer* 2018; 9: 1782–1787.

51. Ghiglione L, Galvez CC, Reig O, et al. Patterns and outcomes related to rapid progressive disease in a cohort of advanced solid tumours treated with immune checkpoint inhibitors (ICIs). *Ann Oncol* 2019; 30: v521–v522.

52. Yang S, Liu X, Bei T, et al. Prevalence of hyperprogressive disease (HPD) mutations and correlations to immune-related biomarkers in a large pan-cancer Chinese cohort. *J Clin Oncol*; 39. Epub ahead of print 2021. DOI: 10.1200/JCO.2021.39.15_suppl.2649.

53. Chen Y, Li X, Liu G, et al. ctDNA Concentration, MIKI67 Mutations and Hyper-Progressive Disease Related Gene Mutations Are Prognostic Markers for Camrelizumab and Apatinib Combined Multiline Treatment in Advanced NSCLC. *Front Oncol* 2020; 10: 1706.

54. Boutin M, De Angelis F, Éthier V, et al. REAL WORLD EFFICACY AND TOXICITY OF ANTI-PD1 TREATMENT IN ELDERLY PATIENTS WITH ADVANCED NON-SMALL CELL LUNG CANCER (NSCLC). *J Geriatr Oncol* 2019; 10: S42–S44.

55. Español-Rego M, Fernández-Martos C, Fernandez MEE, et al. A phase I-II multicenter trial with avelumab plus autologous dendritic cell vaccine in pre-treated mismatch repair-proficient (MSS) metastatic colorectal cancer patients. GEMCAD 16-02 (AVEVAC trial). *Ann Oncol* 2021; 32: S555.

56. Yu S, Hu R, Shi M. Anti-PD-1 antibody monotherapy or anti-PD-1 antibody combination with chemotherapy treated nonsmall cell lung cancer (NSCLC) patients with EGFR mutation: A retrospective analysis. *J Clin Oncol*; 38. Epub ahead of print 2020. DOI: 10.1200/JCO.2020.38.15_suppl.e21691.

57. Raggi D, Giannatempo P, Mariani L, et al. Apache: An open label, randomized, phase 2 study of durvalumab (Durva), alone or in combination with tremelimumab (Treme), in patients (pts) with advanced germ cell tumors (GCT): Results at the end of first stage. *J Clin Oncol*; 36. Epub ahead of print 2018. DOI: 10.1200/JCO.2018.36.15_suppl.4547.

58. Alcaraz Sanchez JJ, Del Rio Valencia JC, Pintado Álvarez A, et al. Atezolizumab in non-small cell lung cancer: Effectiveness and safety real world data study. *Euro J Hosp Pharm Sci Pra* 2021; 28: A53.

59. Nosaki K, Umeyama Y, Toyozawa R, et al. Clinical Background and Response to Chemotherapy in NSCLC Patients with MET Exon14 Skipping Mutation or High MET Gene Copy Number. *J Thorac Oncol* 2018; 13: S896.

60. Lau KS, Liu R, Wong CC, et al. Clinical outcome and toxicity for immunotherapy treatment in metastatic cancer patients. *Ann Palliat Med* 2020; 9: 4446–4457.

61. Rapposelli IG, De Matteis S, Lanuti P, et al. Heterogeneity of Response and Immune System Activity during Treatment with Nivolumab in Hepatocellular Carcinoma: Results from a Single-Institution Retrospective Analysis. *Cancers*; 13.

62. Boileve A, Carlo M, Barthelemy P, et al. Immune checkpoint inhibitors following targeted therapies in MITF family translocation renal cell carcinomas. *Ann Oncol* 2017; 28: v414.

63. Chen JP, Lu WC, Hong RL, et al. Novel immunotherapy combinations for betel-nuts related HNSCC: One institutional experience in Taiwan. *J Clin Oncol*; 38. Epub ahead of print 2020. DOI: 10.1200/JCO.2020.38.15-suppl.e18502.

64. Rubio XM, Aguado C, Sereno M, et al. P1.04-16 Early Antibiotic Use Affects the Efficacy of First Line Immunotherapy in Lung Cancer Patients but Route of Administration Seems to be Decisive. *J Thorac Oncol* 2019; 14: S445.

65. Gunduz S, Ozdogan M, Kırca O, et al. P2.01-57 New Prognostic Markers in Patients with Lung Cancer Treated with Immunoterapy: NLR and PLR. *J Thorac Oncol* 2019; 14: S661–S662.

66. Rubio XM, Cruz P, Sereno M, et al. P2.01-98 Neutrophil-Platelet Score (NPS), a Predictive Systemic Inflammation Score for Pembrolizumab in First Line of Advanced NSCLC Patients. *J Thorac Oncol* 2019; 14: S679.

67. Middleton G, Brock K, Summers Y, et al. Pembrolizumab in performance status 2 patients with non-small cell lung cancer (NSCLC): Results of the PePS2 trial. *Ann Oncol* 2018; 29: viii497.

68. Wrangle JM, Awad MM, Badin FB, et al. Preliminary data from QUILT 3.055: A phase 2 multi-cohort study of N803 (IL-15 superagonist) in combination with checkpoint inhibitors (CPI). *J Clin Oncol*; 39. Epub ahead of print 2021. DOI: 10.1200/JCO.2021.39.15_suppl.2596.

69. Taugner J, Käsmann L, Eze C, et al. Real-world prospective analysis of treatment patterns in durvalumab maintenance after chemoradiotherapy in unresectable, locally advanced NSCLC patients. *Invest New Drugs* 2021; 39: 1189–1196.

70. Bruixola G, Caballero Daroqui J, Cunquero Tomas AJ, et al. Safety and efficacy of nivolumab (nivo) in platinum-refractory recurrent/metastastic head and neck squamous cell (PR R/M HNSCC) patients (pts): Real-life experience. *Ann Oncol* 2018; 29: viii383–viii384.

71. Granados ALO, Caro NL, Pozo JFM, et al. Survival with nivolumab therapy in recurrent/advanced squamous cell head and neck carcinoma. A single center experience. *J Clin Oncol*; 36. Epub ahead of print 2018. DOI: 10.1200/JCO.2018.36.15-suppl.e18032.

72. Plimack ER, Campbell K, Issa JPJ, et al. A Phase II trial of guadecitabine (G) plus atezolizumab (A) in patients with metastatic urothelial carcinoma (UC) progressing after initial checkpoint inhibitor therapy. *Cancer Res*; 81. Epub ahead of print 2021. DOI: 10.1158/1538-7445.Am2021-ct121.

73. Mollica V, Brocchi S, Dall’Olio FG, et al. Tumor Growth Rate Decline despite Progressive Disease May Predict Improved Nivolumab Treatment Outcome in mRCC: When RECIST Is Not Enough. *Cancers* 2021; 13: 3492.

74. Tunali I, Gray JE, Qi J, et al. Novel clinical and radiomic predictors of rapid disease progression phenotypes among lung cancer patients treated with immunotherapy: An early report. *Lung Cancer Amst Neth* 2019; 129: 75–79.

75. Trotier D, Grover P, Park C, et al. Genetic and molecular analysis of solid tumors with hyperprogressive disease after treatment with immunotherapy. *Ann Oncol* 2021; 32: S836–S837.

76. Gomez LG, Gonzalez D, Samson B, et al. P2.04-77 Hyperprogression with Immunotherapy in Metastatic Non-Small Cell Lung Cancer: Hôpital Charles-LeMoyne Experience. *J Thorac Oncol* 2019; 14: S739.

77. Kim SR, Chun SH, Kim JR, et al. The implications of clinical risk factors, CAR index, and compositional changes of immune cells on hyperprogressive disease in non-small cell lung cancer patients receiving immunotherapy. *BMC Cancer* 2021; 21: 19.

78. Lee C, Cheon J, Kim EJ, et al. Characterization of hyperprogressive disease in patients with advanced biliary tract cancer treated with anti-PD-1 inhibitor: A multicenter retrospective study. *J Clin Oncol* 2021; 39: 339–339.

79. Vaidya P, Bera K, Patil PD, et al. Novel, non-invasive imaging approach to identify patients with advanced non-small cell lung cancer at risk of hyperprogressive disease with immune checkpoint blockade. *J Immunother Cancer* 2020; 8: e001343.

80. He S, Feng Y, Lin Q, et al. CT-Based Peritumoral and Intratumoral Radiomics as Pretreatment Predictors of Atypical Responses to Immune Checkpoint Inhibitor Across Tumor Types: A Preliminary Multicenter Study. *Front Oncol*; 11. Epub ahead of print 2021. DOI: 10.3389/fonc.2021.729371.

81. Takahashi R, Shibata E, Higashiyama T, et al. Neutrophil-to-Lymphocyte ratio as a predictive factor for hyperprogressive disease in NSCLC patients treated with immune checkpoint inhibitor. *Ann Oncol* 2019; 30: ix113.

82. Miyama Y, Morikawa T, Miyakawa J, et al. Squamous differentiation is a potential biomarker predicting tumor progression in patients treated with pembrolizumab for urothelial carcinoma. *Pathol Res Pract* 2021; 219: 153364.

83. Han J, Wang Z, Wang Y, et al. Abstract 3247: Dynamic clonality of T cell receptor differentiate atypical progression in NSCLC patients treated with PD-1/PD-L1inhibitors. *Cancer Res* 2019; 79: 3247.

84. Zalcman G, Mazieres J, Greillier L, et al. Second/third-line nivolumab vs nivo plus ipilimumab in malignant pleural mesothelioma: Long-term results of IFCT-1501 MAPS2 phase IIR trial with a focus on hyperprogression (HPD). *Ann Oncol* 2019; 30: v747.

85. Alfieri S, Ferrara R, Calareso G, et al. Evaluating hyperprogressive disease (HPD) in head and neck squamous cell carcinoma (HNSCC) patients treated with immune checkpoint inhibitors (ICI). *Tumori* 2019; 105: 141–142.

86. Paydary K, Moturi KR, Baral B, et al. Hyperprogression on immune checkpoint inhibitors: A single institution, real-world retrospective analysis. *J Clin Oncol* 2020; 38: e15143–e15143.

87. Ferrara R, Lo Russo G, Jachetti E, et al. First-line platinum-based chemotherapy combined with PD-1/PD-l1 inhibitors (ICI) prevents hyperprogression in non-small cell lung cancer (NSCLC) patients by reducing circulating immature neutrophils. *J Immunother Cancer* 2021; 9: A16.

88. Xiao LS, Li QM, Hu CY, et al. Lung metastasis and lymph node metastasis are risk factors for hyperprogressive disease in primary liver cancer patients treated with immune checkpoint inhibitors. *Ann Palliat Med* 2021; 10: 11244–11254.

89. Forschner A, Hilke F-J, Bonzheim I, et al. MDM2, MDM4 and EGFR Amplifications and Hyperprogression in Metastatic Acral and Mucosal Melanoma. *Cancers* 2020; 12: E540.

90. Abbas W, Rao RR, Popli S. Hyperprogression after immunotherapy. *South Asian J Cancer* 2019; 08: 244–246.

91. Costantini A, Corny J, Fallet V, et al. Hyper-progressive disease in patients with advanced non-small cell lung cancer (NSCLC) treated with nivolumab (nivo). *Eur Resp J*; 52.

92. Castello A, Rossi S, Mazziotti E, et al. Hyperprogressive Disease in Patients with Non-Small Cell Lung Cancer Treated with Checkpoint Inhibitors: The Role of 18F-FDG PET/CT. *J Nucl Med Off Publ Soc Nucl Med* 2020; 61: 821–826.

93. Kang Y-K, Reck M, Nghiem P, et al. Assessment of hyperprogression versus the natural course of disease development with nivolumab with or without ipilimumab versus placebo in phase III, randomized, controlled trials. *J Immunother Cancer* 2022; 10: e004273.

94. Choi YJ, Kim T, Kim EY, et al. Prediction model for hyperprogressive disease in non‐small cell lung cancer treated with immune checkpoint inhibitors. *Thorac Cancer* 2020; 11: 2793–2803.

95. Perna M, Scotti V, Muntoni C, et al. Clinico-radiological pattern of response to nivolumab in stage IV NSCL: A real life experience over two years. *J Thorac Oncol* 2018; 13: S110.

96. Ale Tadesse E, Heslin K, Hendawi M, et al. Molecular alterations with hyperprogression in lung cancer patients treated with immune checkpoint inhibitors in a large health system. *J Clin Oncol*; 38. Epub ahead of print 2020. DOI: 10.1200/JCO.2020.38.15_suppl.e15082.

97. Tadesse EA, Heslin K, Hendawi M, et al. Genomic markers associated with hyperprogression in patients with lung cancer treated with immune checkpoint inhibitors. *J Clin Oncol*; 39. Epub ahead of print 2021. DOI: 10.1200/JCO.2021.39.15_suppl.9105.

98. Rimola J, Da Fonseca LG, Sapena V, et al. Radiological response to nivolumab in patients with hepatocellular carcinoma: A multicenter analysis of real-life practice. *Eur J Radiol* 2021; 135: 109484.

99. Reck M, Feng Y, Kim HR, et al. Analysis of tumour hyperprogression (HP) with nivolumab (Nivo) in randomized, placebo (Pbo)-controlled trials. *Ann Oncol* 2019; 30: v486.

100. Colle E, Dalle S, Mortier L, et al. Hyperprogression in ‘real world’ advanced melanoma patients treated by anti-PD1. *Pigm Cell Melanoma Res* 2020; 33: 201.

101. Decatris MP, Thomas JA, Hayes M, et al. Exploratory analysis of factors associated with hyperprogression in advanced non-small cell lung cancer (NSCLC) treated with PD1/PDL1 inhibitors. *J Clin Oncol*; 38. Epub ahead of print 2020. DOI: 10.1200/JCO.2020.38.15_suppl.e21634.

102. Dey S, Ali MAM, Gunchick V, et al. Hyperprogression in cancer patients on immunotherapeutic agents. *J Clin Oncol*; 38. Epub ahead of print 2020. DOI: 10.1200/JCO.2020.38.15_suppl.3575.

103. Ruiz-Patiño A, Arrieta O, Cardona AF, et al. Immunotherapy at any line of treatment improves survival in patients with advanced metastatic non-small cell lung cancer (NSCLC) compared with chemotherapy (Quijote-CLICaP). *Thorac Cancer* 2020; 11: 353–361.

104. Yilmaz M. Atypical response patterns in metastatic melanoma and renal cell carcinoma patients treated with nivolumab: A single center experience. *J Oncol Pharm Pract Off Publ Int Soc Oncol Pharm Pract* 2021; 27: 1106–1111.

105. Jin T, Zhang Q, Jin Q-F, et al. Anti-PD1 checkpoint inhibitor with or without chemotherapy for patients with recurrent and metastatic nasopharyngeal carcinoma. *Transl Oncol* 2021; 14: 100989.

106. Matos I, Martin-Liberal J, Hierro C, et al. Incidence and clinical implications of a new definition of hyperprogression (HPD) with immune checkpoint inhibitors (ICIs) in patients treated in phase 1 (Ph1) trials. *J Clin Oncol* 2018; 36: 3032–3032.

107. Lee B, Lee G, Kwon WS, et al. Hyperprogressive disease after two cycles of immunotherapy in HER-2 positive metastatic gastric cancer. *Mol Cancer Ther*; 18. Epub ahead of print 2019. DOI: 10.1158/1535-7163.Targ-19-b049.

108. Giusti R, Mazzotta M, Filetti M, et al. CDKN2A/B gene loss and MDM2 alteration as a potential molecular signature for hyperprogressive disease in advanced NSCLC: A next-generation-sequencing approach. *J Clin Oncol*; 37. Epub ahead of print 2019. DOI: 10.1200/JCO.2019.37.15_suppl.e20628.

109. Fricke J, Mambetsariev I, Pharaon R, et al. Alterations in STK11 to limit response to immune checkpoint inhibitors in lung cancer. *J Clin Oncol*; 38. Epub ahead of print 2020. DOI: 10.1200/JCO.2020.38.15-suppl.e21503.

110. Jang T. P75.13 Hyperprogressive Disease in Non-Small Cell Lung Cancer on pd-1 Inhibitor. *J Thorac Oncol* 2021; 16: S579.

111. Ayers KL, Mullaney T, Zhou X, et al. Analysis of Real-World Data to Investigate the Impact of Race and Ethnicity on Response to Programmed Cell Death-1 and Programmed Cell Death-Ligand 1 Inhibitors in Advanced Non-Small Cell Lung Cancers. *The Oncologist* 2021; 26: e1226–e1239.

112. Petrova MP, Donev IS, Radanova MA, et al. Sarcopenia and high NLR are associated with the development of hyperprogressive disease after second-line pembrolizumab in patients with non-small-cell lung cancer. *Clin Exp Immunol* 2020; 202: 353–362.

113. Feng Y, Nghiem P, Zwirtes R, et al. Evaluating the occurrence of early tumor progression (ETP) in patients with gastric cancer treated with nivolumab versus placebo. *J Immunother Cancer*; 6. Epub ahead of print 2018. DOI: 10.1186/s40425-018-0423-x.

114. Middleton G, Brock K, Savage J, et al. Pembrolizumab in patients with non-small-cell lung cancer of performance status 2 (PePS2): a single arm, phase 2 trial. *Lancet Respir Med* 2020; 8: 895–904.

115. Kawabata R, Kurokawa Y, Hagi T, et al. Real-world efficacy and biomarker of nivolumab for advanced gastric cancer. *J Clin Oncol*; 38. Epub ahead of print 2020. DOI: 10.1200/JCO.2020.38.4_suppl.388.

116. Aoki M, Shoji H, Imazeki H, et al. The hyperprogressive disease during nivolumab treatment or irinotecan treatment in patients with advanced gastric cancer. *J Clin Oncol*; 37, https://www.embase.com/search/results?subaction=viewrecord&id=L627163795&from=export (2019).

117. Zuazo M, Arasanz H, Chocarro L, et al. 1354P Comparison of different hyperprogressive disease criteria in non-small cell lung cancer patients treated with immunotherapy and correlation with somatic mutations in driver genes. *Ann Oncol* 2021; 32: S1028.

118. Arasanz H, Zuazo M, Martínez Aguillo M, et al. A burst of highly differentiated CD4 TL identifies a subset of fast progressors, and correlates with hyperprogressive disease in NSCLC patients treated with ICI. *Ann Oncol* 2019; 30: v521.

119. Ayala De Miguel P, López Gallego J, Gorospe García I, et al. Hyperprogressive disease in patients with advanced non-small cell lung cancer treated with immune checkpoint inhibitors. *Ann Oncol* 2019; 30: xi25.

120. Economopoulou P, Spathas NS, Papaxoinis G, et al. Clinical implications of hyperprogression with immune checkpoint inhibitors in patients with head and neck squamous cell carcinoma (HNSCC). *J Clin Oncol*; 37. Epub ahead of print 2019. DOI: 10.1200/JCO.2019.37.15-suppl.6034.

121. Psyrri A, Gavrielatou N, Spathis A, et al. Predictive biomarkers for response to nivolumab in head and neck squamous cell carcinoma (HNSCC) (NCT#03652142). *J Clin Oncol*; 37. Epub ahead of print 2019. DOI: 10.1200/JCO.2019.37.15-suppl.6060.

122. Lo Russo G, Moro M, Sommariva M, et al. Antibody-Fc/FcR Interaction on Macrophages as a Mechanism for Hyperprogressive Disease in Non-small Cell Lung Cancer Subsequent to PD-1/PD-L1 Blockade. *Clin Cancer Res Off J Am Assoc Cancer Res* 2019; 25: 989–999.

123. Ferrara R, Mezquita L, Texier M, et al. Comparison of Fast-Progression, Hyperprogressive Disease, and Early Deaths in Advanced Non-Small-Cell Lung Cancer Treated With PD-1/PD-L1 Inhibitors or Chemotherapy. *JCO Precis Oncol* 2020; 4: 829–840.

124. Abbar B, De Castelbajac V, Gougis P, et al. Definitions, outcomes, and management of hyperprogression in patients with non-small-cell lung cancer treated with immune checkpoint inhibitors. *Lung Cancer Amst Neth* 2021; 152: 109–118.

125. Ferrara R, Mezquita L, Texier M, et al. Fast-progression (FP), hyper-progression (HPD) and early deaths (ED) in advanced non-small cell lung cancer (NSCLC) patients (pts) upon PD-(L)-1 blockade (IO). *J Clin Oncol*; 37. Epub ahead of print 2019. DOI: 10.1200/JCO.2019.37.15_suppl.9107.

126. Ferrara R, Caramella C, Texier M, et al. Hyperprogressive disease (HPD) is frequent in non-small cell lung cancer (NSCLC) patients (pts) treated with anti PD1/PD-L1 monoclonal antibodies (IO). *Ann Oncol* 2017; 28: v464–v465.

127. Ferrara R, Caramella C, Texier M, et al. Hyperprogressive disease (HPD) is frequent in non-small cell lung cancer (NSCLC) patients (pts) treated with anti PD1/PD-L1 agents (IO). *J Thorac Oncol* 2017; 12: S1843.

128. Giusti R, Filetti M, Mazzotta M, et al. CDKN2A/B gene loss and MDM2 alteration as a potential molecular signature for hyperprogressive disease in advanced NSCLC: A nextgeneration-sequencing approach. *Tumori* 2019; 105: 87–88.

129. De Morais ALG, Cardenas JM, De Miguel Luken MJ, et al. Comparative assessment of different radiological criteria to identify paradoxical hyperprogression (HPD) to IO drugs. *J Clin Oncol*; 38. Epub ahead of print 2020. DOI: 10.1200/JCO.2020.38.15_suppl.e15229.

130. Lu WC, Hong RL. Immunotherapy combinations for betel-nuts related HNSCC: one institutional experiences in Taiwan. *J Immunother Cancer*; 7. Epub ahead of print 2019. DOI: 10.1186/s40425-019-0763-1.

131. Chen JP, Chang JY, Kuo SH, et al. Possible CDK4/6 inhibitor use in betel-nuts related head and neck squamous cell carcinoma(HNSCC). *J Clin Oncol*; 36. Epub ahead of print 2018. DOI: 10.1200/JCO.2018.36.15-suppl.e18030.

132. Hwang I, Park I, Yoon SK, et al. Hyperprogressive disease (HPD) in genitourinary (GU) cancer patients treated with PD-1/PD-L1 inhibitors. *J Clin Oncol*; 37, https://www.embase.com/search/results?subaction=viewrecord&id=L627164125&from=export (2019).

133. Kanjanapan Y, Day D, Wang L, et al. Hyperprogressive disease (HPD) in earlyphase immunotherapy (IO) trials. *J Clin Oncol*; 36. Epub ahead of print 2018. DOI: 10.1200/JCO.2018.36.15_suppl.3063.

134. Tan TJY, Cescon DW, Wang L, et al. Hyperprogressive disease in advanced triple-negative breast cancer (aTNBC) treated with immunotherapy (IO). *J Clin Oncol* 2019; 37: 1086–1086.

135. Al-ezzi EM, Alshammari K, Kanjanapan Y, et al. 1710P - Impact of immunotherapy and targeted therapy on tumour growth rate in sarcoma. *Ann Oncol* 2019; 30: v700–v701.

136. Chon H, Kim CG, Yoon S, et al. Hyperprogressive disease during PD-1 blockade in patients with advanced hepatocellular carcinoma. *J Clin Oncol*; 38. Epub ahead of print 2020. DOI: 10.1200/JCO.2020.38.4_suppl.550.

137. Kim Y, Kim CH, Lee S, et al. OA14.07 Clinical and Genetic Characterization of Hyperprogression Based on Volumetry in Advanced NSCLC Treated with Immunotherapy. *J Thorac Oncol* 2019; 14: S245.

138. Kim KH, Hur JY, Ku BM, et al. P14.25 Immune Cell Profiling of Hyperprogressive Disease in Patients with Non-Small Cell Lung Cancer Treated with Anti-PD-1/PD-L1 Antibodies. *J Thorac Oncol* 2021; 16: S340.

139. Kim Y, Kim CH, Lee HY, et al. Comprehensive Clinical and Genetic Characterization of Hyperprogression Based on Volumetry in Advanced Non–Small Cell Lung Cancer Treated With Immune Checkpoint Inhibitor. *J Thorac Oncol* 2019; 14: 1608–1618.

140. Kim Y, Kim CH, Kim HS, et al. Hyperprogression after immunotherapy: Clinical implication and genomic alterations in advanced non-small cell lung cancer patients (NSCLC). *J Clin Oncol*; 36. Epub ahead of print 2018. DOI: 10.1200/JCO.2018.36.15-suppl.9075.

141. Ku BM, Kim Y, Lee KY, et al. Tumor infiltrated immune cell types support distinct immune checkpoint inhibitor outcomes in patients with advanced non-small cell lung cancer. *Eur J Immunol* 2021; 51: 956–964.

142. Song C, Park H, Lee HY, et al. Evaluation of Response to Immune Checkpoint Inhibitors Using a Radiomics, Lesion-Level Approach. *Cancers* 2021; 13: 6050.

143. Lee JC, Choi C, Ji W, et al. P2.01-46 The Efficacy and Safety of 2nd-Line Nivolumab for Non-Small Cell Lung Cancer in Real-World Practice with Emphasis on Hyperprogession. *J Thorac Oncol* 2019; 14: S657.

144. Kim S, Kim SY, Kang JH. A new pattern called Hyperprogression when using Immune checkpoint blockers in real world. *Cancer Res*; 79. Epub ahead of print 2019. DOI: 10.1158/1538-7445.Sabcs18-5004.

145. Kim S, Kang JH, Chun SH, et al. Clinical implication of inflammation-based serologic biomarkers and tissue biomarkers on hyperprogression in NSCLC patients receiving immune checkpoint blockers in real world. *J Clin Oncol*; 37. Epub ahead of print 2019. DOI: 10.1200/JCO.2019.37.15_suppl.e20633.

146. Kim J, Kim S, Kang JH, et al. Clinical implication of multiplex IHC and serologic biomarkers on hyperprogression in NSCLC patients receiving immune checkpoint blockers in real world. *Ann Oncol* 2019; 30: v620.

147. Kim S, Kim J, Hong S, et al. Hyperprogression and Pseudoprogression in Patients with Non-Small Cell Lung Cancer on Checkpoint Blocking Immunotherapy. *J Thorac Oncol* 2018; 13: S888–S889.

148. Kim S, Chun SH, Kim SY, et al. Multiplex immunohistochemistryaccurately defines the immune compositional change of tumor microenvironment to predict hyperprogressivedisease. *Cancer Res*; 80. Epub ahead of print 2020. DOI: 10.1158/1538-7445.Am2020-3876.

149. Klemen ND, Hwang S, Bradic M, et al. 1527MO Biomarkers of response and hyperprogression in patients with sarcoma treated with checkpoint blockade. *Ann Oncol* 2021; 32: S1114–S1115.

150. Zhong H, Li J, Xiang C, et al. The genomic characterization of different progression patterns in advanced lung cancer (LC) patients upon immune checkpoint inhibitor (ICI) treatment. *J Clin Oncol*; 38. Epub ahead of print 2020. DOI: 10.1200/JCO.2020.38.15_suppl.e21653.

151. Ferrara R, Russo GL, Signorelli D, et al. Circulating and tumor-associated neutrophil subtypes discriminate hyperprogressive disease (HPD) from conventional progression (PD) upon immune checkpoint inhibitors (ICI) in advanced nonsmall cell lung cancer (NSCLC) patients (pts) and in vivo models. *J Clin Oncol*; 38. Epub ahead of print 2020. DOI: 10.1200/JCO.2020.38.15-suppl.9547.

152. Farè E, Sdao S, Damian S, et al. Hyperprogression during immuno-checkpoint inhibitors (ICIs): A clinically significant problem? *Ann Oncol* 2018; 29: viii430.

153. Alfieri S, Ferrara R, Calareso G, et al. Hyperprogressive disease (HPD) in head and neck squamous cell carcinoma (HNSCC) patients treated with immune checkpoint inhibitors (ICI). *J Clin Oncol* 2019; 37: 6029–6029.

154. Ferrara R, Facchinetti F, Calareso G, et al. Hyperprogressive disease (HPD) upon first-line PD-1/PD-L1 inhibitors (ICI) as single agent or in combination with platinum-based chemotherapy in non-small cell lung cancer (NSCLC) patients (pts). *Ann Oncol* 2020; 31: S826.

155. Lo Russo G, Signorelli D, Proto C, et al. Hyperprogressive disease in advanced non-small cell lung cancer patients treated with immunotherapy. *Tumori* 2019; 105: 94.

156. Ferrara R, Jachetti E, Calareso G, et al. Immunometabolism of circulating neutrophils in hyperprogressive disease (HPD) upon first-line PD-1/PD-L1 inhibitors (ICI) alone or in combination with platinum-based chemotherapy (PCT) in non-small cell lung cancer (NSCLC) patients (pts). *Cancer Res*; 81.

157. de Braud F, Di Nicola M, Damian S, et al. Is “hyper-progression” a relevant clinical item for patient with solid tumours candidate to check-point inhibitor treatment? *Ann Oncol* 2018; 29: iii5.

158. Lo Russo G, Signorelli D, Proto C, et al. Hyperprogressive Disease in Advanced Non-Small Cell Lung Cancer Patients Treated with Immune Checkpoint Inhibitors. *J Thorac Oncol* 2019; 14: S245–S245.

159. Sozzi G, Sommariva M, Moro M, et al. PD-1 Blockade Promotes Hyperprogressive Disease in NSCLC Through Macrophages Activation via Antibody-Fc/FcR Interaction. *J Thorac Oncol* 2018; 13: S368.

160. Suarez C, Morales-Barrera R, Garcia-Ruiz A, et al. Hyperprogressive disease in patients with metastatic genitourinary tumors treated with immune checkpoint inhibitors. *J Clin Oncol*; 37.

161. Freixinos VR, Garcia A, Fasani R, et al. Immune profile and outcomes of patients (pts) with gynecological malignancies (GYN) enrolled in early phases immunotherapy (IO) trials. *J Clin Oncol*; 36. Epub ahead of print 2018. DOI: 10.1200/JCO.2018.36.15_suppl.5595.

162. Garcia IM, Ruiz AG, Martin-Liberal J, et al. Refining criteria of hyperprogression (HPD) with immune checkpoint inhibitors (ICIs) to improve clinical applicability. *Ann Oncol* 2018; 29: 653–653.

163. Ortega Franco A, Plana M, Braña I, et al. Does hyper-progression exist among head and neck cancer patients treated with immunotherapy? *Ann Oncol* 2017; 28: v379.

164. Nakamoto R, Zaba L, Rosenberg J, et al. Imaging characteristics and diagnostic performance of 18F-FDG PET/CT for melanoma patients who demonstrate hyperprogressive disease when treated with immunotherapy. *J Nucl Med*; 61, https://www.embase.com/search/results?subaction=viewrecord&id=L633250248&from=export (2020).

165. Petrova M, Eneva M, Arabadjiev JI, et al. High neutrophil to lymphocyte ratio as a predictor for hyperprogressive disease in patients with metastatic non-small cell lung cancer treated with pembrolizumab as a second line. *J Clin Oncol*; 38. Epub ahead of print 2020. DOI: 10.1200/JCO.2020.38.15-suppl.e21546.

166. Necchi A, Mariani L, Raggi D, et al. APACHE: An open label, randomized, phase II study of Durvalumab (Durva), alone or in combination with Tremelimumab (Treme), in patients (pts) with advanced germ cell tumors (GCT): Results at the end of first stage. *Cancer Res*; 78. Epub ahead of print 2018. DOI: 10.1158/1538-7445.Am2018-ct102.

167. Refae S, Ebran N, Gal J, et al. Host immunogenetics and hyperprogression under PD1/PD-L1 checkpoint inhibitors. *Cancer Res*; 78. Epub ahead of print 2018. DOI: 10.1158/1538-7445.Am2018-4548.

168. Simões Da Rocha PF, Ripoll E, Corbera A, et al. Radiological identification of rapid progressions in advanced NSCLC patients treated with nivolumab. *Ann Oncol* 2018; 29: viii534.

169. Arrieta OG, Ruiz-Patiño A, Cardona AF, et al. P1.04-81 Characterization of Hispanic Patients Who Experienced Hyperprogression During Treatment for Advanced NSCLC with Immunotherapy. *J Thorac Oncol* 2019; 14: S474.

170. Arrieta O, Ruiz-Patiño A, Cardona AF, et al. P2.23 Characterization of Hispanic Patients Who Experienced Hyperprogression During Treatment for Advanced NSCLC with Immunotherapy. *J Thorac Oncol* 2019; 14: S1194–S1195.

171. Togashi Y, Kamada T, Sasaki A, et al. Clinicopathological, genomic and immunological features of hyperprogressive disease during PD-1 blockade in gastric cancer patients. *J Clin Oncol*; 36. Epub ahead of print 2018. DOI: 10.1200/JCO.2018.36.15-suppl.4106.

172. Sasaki A, Nakamura Y, Mishima S, et al. Predictive factor of hyperprogressive disease during nivolumab in patients with advanced gastric cancer. *Ann Oncol* 2018; 29: vii56.

173. Scheiner B, Kirstein MM, Hucke F, et al. PD-1 targeted immunotherapy in advanced hepatocellular carcinoma: Efficacy and safety data from an international multicenter real-world cohort. *Z Gastroenterol* 2019; 57: e153–e154.

174. Sugimoto N, Otsuka T, Hasegawa A, et al. Hyperprogressive disease (HPD) during nivolumab (Nivo) or irinotecan (IRI) as salvage line in patients with metastatic gastric cancer (MGC). *J Clin Oncol*; 37, https://www.embase.com/search/results?subaction=viewrecord&id=L627163875&from=export (2019).

175. Sunakawa Y, Takahashi Y, Inoue E, et al. Interim analysis of an observational/translational study for nivolumab treatment in advanced gastric cancer: JACCRO GC-08 (DELIVER trial). *Ann Oncol* 2019; 30: v314.

176. Ishiguro A, Inoue E, Sakamoto Y, et al. O1-4 Survival time of nivolumab treatment in advanced gastric cancer from real-world data of the DELIVER trial (JACCRO GC-08). *Ann Oncol* 2021; 32: S285.

177. Sunakawa Y, Sakamoto Y, Inoue E, et al. Updated analysis of DELIVER trial (JACCRO GC-08): A large observational/translational study of nivolumab treatment in advanced gastric cancer. *Ann Oncol* 2020; 31: S242.

178. Hagi T, Kurokawa Y, Kawabata R, et al. Multicentre biomarker cohort study on the efficacy of nivolumab treatment for gastric cancer. *Br J Cancer* 2020; 123: 965–972.

179. Taugner J, Käsmann L, Eze C, et al. Real-world analysis of treatment patterns and efficacy of durvalumab maintenance after chemoradiotherapy in NSCLC patients. *J Thorac Oncol* 2021; 16: S742.

180. Taugner J, Käsmann L, Eze C, et al. Treatment patterns and efficacy of durvalumab maintenance after CRT in real-world NSCLC patients. *Radiother Oncol* 2021; 161: S968.

181. Berge DT, Hurkmans D, Den Besten I, et al. P1.09-21 Tumor Responses Based on Tumor Growth Rate During PD-1 Inhibitor Therapy in Advanced Non-Small-Cell Lung Cancer Patients. *J Thorac Oncol* 2019; 14: S504.

182. Tunali I, Gray J, Abdullah M, et al. Epidemiologic and radiomic analysis of hyperprogressers of lung cancer patients treated with immunotherapy. *J Thorac Oncol* 2017; 12: S2386.

183. Patil P, Bera K, Vaidya P, et al. Pre-Therapy Radiomic Features Can Distinguish Hyperprogression from Other Response Patterns to PD1/PD-L1 Inhibitors in NSCLC. *J Thorac Oncol* 2018; 13: S737.

184. Ji Z, Cui Y, Peng Z, et al. Use of Radiomics to Predict Response to Immunotherapy of Malignant Tumors of the Digestive System. *Med Sci Monit Int Med J Exp Clin Res* 2020; 26: e924671.

185. Lu Z, Zou J, Hu Y, et al. Serological Markers Associated With Response to Immune Checkpoint Blockade in Metastatic Gastrointestinal Tract Cancer. *JAMA Netw Open* 2019; 2: e197621.

186. Kamada T, Togashi Y, Tay C, et al. PD-1+ regulatory T cells amplified by PD-1 blockade promote hyperprogression of cancer. *Proc Natl Acad Sci* 2019; 116: 9999–10008.

187. Ji Z, Peng Z, Gong J, et al. Hyperprogression after immunotherapy in patients with malignant tumors of digestive system. *BMC Cancer* 2019; 19: 705.

188. Suzuki T, Aoki M, Shirasu H, et al. Hyperprogressive disease during nivolumab chemotherapy in metastatic gastric cancer: Multicenter retrospective study in Japan. *J Clin Oncol*; 38. Epub ahead of print 2020. DOI: 10.1200/JCO.2020.38.4_suppl.377.

189. Guiard E, Baldini C, Pobel C, et al. Radiological patterns of tumour progression in patients treated with a combination of immune checkpoint blockers and antiangiogenic drugs. *Eur J Cancer* 2022; 167: 42–53.

190. Saâda-Bouzid E, Defaucheux C, Karabajakian A, et al. Hyperprogression during anti-PD-1/PD-L1 therapy in patients with recurrent and/or metastatic head and neck squamous cell carcinoma. *Ann Oncol* 2017; 28: 1605–1611.

191. Kadowaki S, Aoki M, Suzuki T, et al. Association of disease progression pattern during third-line chemotherapy with nivolumab with poor prognosis in advanced gastric cancer: A multicenter retrospective study in Japan. *J Clin Oncol*; 40. Epub ahead of print 2022. DOI: 10.1200/JCO.2022.40.4-suppl.258.

192. Kubota Y, Yoshimura K, Hamada K, et al. Rare Nivolumab-associated Super Hyper Progressive Disease in Patients With Advanced Gastric Cancer. *In Vivo* 2021; 35: 1865–1875.

193. Sugimoto N, Ohtsuka T, Fujiishi K, et al. Hyperprogression during nivolumab (Nivo) or irinotecan (IRI) as salvage-line in patients with metastatic gastric cancer. *Ann Oncol* 2018; 29: vii55–vii56.
